# Design and in silico evaluation of PV-GNProtecta: an mRNA multi-epitope vaccine candidate against Puumala virus using immunoinformatics and structural analysis

**DOI:** 10.1186/s44342-026-00081-8

**Published:** 2026-10-12

**Authors:** Tanvir Hossain, Abdullah Al Mahmud, Maisha Afrin, Sakibur Rahman, Shaheda Siddiqa Tahia, Ahmad Abdullah Mahdeen, Md. Habib Ullah Masum

**Affiliations:** 1 Department of Microbiology, Noakhali Science and Technology University, Noakhali, 3814 Bangladesh; 2 Department of Biochemistry and Molecular Biology, University of Chittagong, Chittagong, 4331 Bangladesh; 3 Department of Microbiology, Notre Dame University Bangladesh (NDUB), Dhaka, 1000 Bangladesh; 4 Department of Genomics and Bioinformatics, Faculty of Biotechnology and Genetic Engineering, Chattogram Veterinary and Animal Sciences University, Khulshi, 4225 Bangladesh

**Keywords:** Nephropathia epidemica (NE), MRNA vaccine, Epitope, Multi-epitope vaccine, Hantavirus, Reverse vaccinology (RV), Molecular dynamics simulation

## Abstract

Puumala virus (PUUV), the principal causative agent of nephropathia epidemica (NE), poses a significant public health threat due to its zoonotic transmission, renal complications, and the lack of an approved vaccine. In this study, an immunoinformatics-driven mRNA multi-epitope vaccine, PV-GNProtecta, was designed using conserved regions of the viral glycoprotein (G) and nucleoprotein (N). Highly antigenic, non-allergenic, and non-toxic T-cell and B-cell epitopes were identified and assembled with appropriate adjuvants to enhance immunogenicity. Population coverage analysis suggested broad HLA responsiveness across diverse populations. The vaccine construct exhibited favorable physicochemical properties, including stability, solubility, and antigenicity. Structural validation predicted high model quality, with 95.5% of residues located in favored Ramachandran regions and an ERRAT score of 91.4. Molecular docking and MM-GBSA analyses predicted favorable computational interactions between PV-GNProtecta and TLR4, while a 100 ns molecular dynamics simulation provided computational insights into the dynamic behavior of the vaccine-TLR4 complex. Codon optimization suggested compatibility of the designed sequence with an *Escherichia coli*-based expression framework. Immune simulation predicted potential humoral and cellular immune response patterns, including changes in IgG, IFN-γ, and IL-2 levels. The designed mRNA sequence exhibited favorable predicted secondary structural characteristics; however, further experimental evaluation is required to assess translational efficiency and stability. Although this study is limited to computational analyses and requires experimental validation, the findings suggest that PV-GNProtecta may represent a potential candidate for further investigation against PUUV infection.

**Supplementary Information:**

The online version contains supplementary material available at https://doi.org/10.1186/s44342-026-00081-8.

## Introduction

Nephropathia epidemica (NE) is attributed to Puumala virus (PUUV), a naturally occurring form of hemorrhagic fever with renal syndrome (HFRS) in humans, which is a zoonotic infectious disease and generally considered a mild-to-moderate form of hantavirus infection within the HFRS spectrum [1–4]. PUUV (*Puumala orthohantavirus*) is classified within the genus *Orthohantavirus*, family *Hantaviridae*, and order *Bunyavirales*, as defined by current ICTV taxonomy, and it was previously grouped under the family *Bunyaviridae* prior to taxonomic reclassification [5, 6].

PUUV infection presents with highly variable outcomes, ranging from subclinical cases (70–80%) to severe HFRS, and carries a fatality rate of about 0.1% [7]. PUUV infection commonly presents with sudden fever, headache, back pain, nausea, gastrointestinal symptoms, thrombocytopenia, hematuria, and varying degrees of acute kidney impairment [1, 8]. In some patients, additional manifestations such as blurred vision, dizziness, insomnia, respiratory complications, oliguria, and subsequent polyuria during recovery may also occur [2, 9]. Although PUUV infection is generally considered mild, severe complications can occur in a subset of patients, including acute kidney failure, hypotension, shock, pulmonary edema, Guillain-Barré syndrome, respiratory distress, disseminated intravascular coagulopathy (DIC), myocarditis, encephalitis, pituitary hemorrhage, seizures, and, in rare cases, multiorgan failure or fatal cerebral hemorrhage [2, 3, 10, 11].

The first identification of PUUV occurred in the early 1980s when the virus was isolated from bank voles (*Myodes glareolus*) in the Puumala region of Finland [2]. Between July 1989 and June 1996, Finland reported 6701 PUUV infection cases confirmed via serological assays, with the highest incidence in the Itä-Savo district and a mortality rate below 0.1%, and later from 1995 to 2008, 22,681 cases were recorded with an average annual incidence of 31 per 100,000 population, resulting in 13 deaths and 52% of patients requiring hospitalization [12, 13]. PUUV accounts for approximately 93% of all notified hantavirus infections in Germany and caused a major outbreak in 2007 in Baden-Württemberg with 1089 reported human cases, while the highest annual number of PUUV infections in Germany was recorded in 2010 since hantavirus surveillance began in 2001 [14, 15]. Also in 2007, Sweden’s Västerbotten County experienced a surprisingly severe outbreak of PUUV infection, with NE rates hitting 313 cases per 100,000 residents [16, 17]. Between 2001 and 2017, a total of 12,148 hantavirus disease cases were recorded, of which 9714 (approximately 97.41%) were attributed to PUUV infection [18]. In 2019, Europe recorded over 4000 hantavirus cases (0.8 per 100,000), 98% caused by PUUV, with Finland and Germany making up 69% of the total [19]. From 2018 to 2025, Hungary recorded 51 lab-confirmed hantavirus infections, 20 of which were specifically linked to the PUUV [20]. In Finland, between 560 and 3800 cases of NE are reported each year, though the actual number is believed to be nearly six times higher [21].

PUUV is a spherical enveloped pathogen characterized by a segmented negative-sense single-stranded RNA (ssRNA) genome [5, 21]. The PUUV possesses a tripartite genome: the L segment (~ 6.5 kb) encodes a ~ 2156-amino-acid L protein (RNA-dependent RNA polymerase) with replicase, transcriptase, and endonuclease functions; the M segment (~ 3.7 kb) encodes a ~ 1148-amino-acid glycoprotein precursor that is co-translationally cleaved into Gn (70 kDa) and Gc (55 kDa); and the S segment (~ 1.8 kb) encodes a ~ 433-amino-acid nucleocapsid (N) protein [2, 5, 22, 23]. Gc is the protein that mediates fusion by the identification of a peptide of fusion, whereas Gn serves as a folding chaperone for Gc and controls the time of fusion; both are necessary for the virus to adhere as well as enter host cells [2, 24]. Gn and Gc form the principal antigenic surface of PUUV and are major targets of neutralizing antibodies, contributing to protective immunity and vaccine relevance [25–27]. The N protein plays multiple roles in viral genome transport, replication, and assembly through its interactions with host cellular proteins, while also acting as a highly immunogenic viral component in both humans and animal models [28, 29]. The N protein is highly conserved among *orthohantaviruses* and elicits a strong early antibody response during acute infection, making it an important target for both diagnostic applications and vaccine development [30, 31].

Various vaccine platforms are available, each possessing distinct advantages and drawbacks. For example, live attenuated vaccines trigger strong, long-lasting immunity but can be risky for immunocompromised individuals due to possible reversion to virulence; inactivated vaccines are safer and more stable, though they usually require multiple doses; subunit vaccines use only key antigens, lowering the chance of side effects, but often need adjuvants and booster shots to achieve durable protection [32–35]. Moreover, DNA vaccines offer rapid, stable, and cost-effective production with long-lasting immunity, but they often show weak immunogenicity in humans and face delivery challenges [34, 36]. In contrast, the key advantage of mRNA vaccines lies in their rapid design and production, enabling swift responses to emerging infections and pandemic threats without the need to culture live pathogens [37, 38]. In recognition of these advances, the 2023 Nobel Prize in Physiology or Medicine honored Katalin Karikó and Drew Weissman for their groundbreaking contributions to nucleoside engineering, a key innovation that enabled the successful development of mRNA vaccines against COVID-19 [39]. Recent developments in bioinformatics have improved biopharmaceutical design by enabling rapid prediction of protein features, structural modeling, and immunogenicity assessment. These computational approaches accelerate the identification and optimization of therapeutic candidates while reducing time and experimental costs; however, challenges related to prediction accuracy, biological complexity, and the need for experimental validation remain important considerations [40]. Reverse vaccinology (RV) applies bioinformatics and immunoinformatics to screen microbial genomes for conserved, immunogenic epitopes, enabling faster, safer, and cost-effective vaccine development compared to traditional pathogen culturing, a slow and labor-intensive process [41, 42]. Vaccine development against PUUV infection has included inactivated whole-virus vaccines, recombinant subunit vaccines, virus-like particle (VLP)-based approaches, and DNA vaccines [43–45]. Among these, inactivated and DNA vaccine candidates have demonstrated promising immunogenicity in preclinical studies [44–46]. Currently, no vaccine has been authorized by the U.S. Food and Drug Administration (FDA) for PUUV infection [9, 47].

Several studies have suggested the potential of multi-epitope vaccine approaches using immunoinformatics-based strategies. Khan et al. developed a multi-epitope vaccine candidate against SARS-CoV-2 by integrating epitope prediction, antigenicity assessment, molecular docking, molecular dynamics simulation, and immune response analysis, demonstrating the feasibility of computational vaccine design approaches [48]. Similarly, Peele et al. designed a multi-epitope vaccine construct containing predicted B-cell and T-cell epitopes and evaluated its structural and immunological properties through computational analyses [49]. These studies highlighted the potential of combining multiple immune targets within a single vaccine construct; however, their findings remained primarily dependent on computational predictions and required experimental validation to confirm immunogenicity and protective efficacy.

However, despite these advances, several challenges remain in current multi-epitope vaccine development strategies. Computational vaccine designs are dependent on prediction algorithms, reference databases, structural modeling assumptions, and available biological information, which may influence the reliability of predicted vaccine candidates. In addition, variations in epitope selection criteria, antigen conservation analysis, population coverage evaluation, and structural validation approaches can affect the effectiveness of computational vaccine design. Recent studies have emphasized that although immunoinformatics approaches accelerate multi-epitope vaccine development, comprehensive experimental validation remains essential for confirming biological relevance and protective potential [50].

Despite previous vaccine development efforts, including inactivated whole-virus vaccines, recombinant subunit vaccines, virus-like particle (VLP)-based approaches, and DNA vaccine candidates, no PUUV vaccine has currently received regulatory approval. An integrated mRNA multi-epitope vaccine strategy targeting conserved regions of both PUUV glycoproteins (Gn/Gc) and nucleocapsid protein remains insufficiently explored. A comprehensive framework combining conserved antigen identification, multi-epitope vaccine construction, structural evaluation, molecular docking, molecular dynamics simulation, and immune response prediction for PUUV has not been extensively investigated. Therefore, this study aimed to design and evaluate the PV-GNProtecta vaccine candidate using an integrated immunoinformatics workflow involving conserved antigen identification, epitope prediction, structural modeling, molecular docking, molecular dynamics simulation, and immune response prediction. The findings provide computational insights into the potential characteristics of PV-GNProtecta while recognizing the necessity of experimental validation for future assessment.

## Materials and methods

### Acquisition and characterization of viral protein sequences

The UniProt protein database (https://www.uniprot.org/; accessed on 15 July 2025) was used for obtaining amino acid sequences of the target proteins from PUUV of G (accession number: P41266) and N (accession numbers: P41270) [51]. The G and N proteins were selected due to their key roles in PUUV infection and immune recognition. The G protein mediates viral attachment and entry and is a major target of neutralizing antibodies, whereas the highly conserved N protein contributes to viral replication and induces strong immune responses. Compared with the intracellular RdRp/L protein, G and N provide more suitable targets for antibody and cellular immune responses. A comparative evaluation of these proteins is provided in Supplementary Table S14.

### Physicochemical and structural characterization of selected proteins

The selected PUUV G and N proteins were characterized for signal peptide presence, transmembrane regions, and subcellular localization. Signal peptide prediction was performed using SignalP 6.0 (https://services.healthtech.dtu.dk/services/SignalP-6.0/; accessed on 15 September 2026) with the organism group selected as Eukarya and the default prediction model [52]. Transmembrane topology was predicted using DeepTMHMM version 1.0.57 (accessed on 15 September 2026) using the default settings [53]. Subcellular localization information was obtained from UniProt (https://www.uniprot.org/; accessed on 15 September 2026) based on available curated annotations [54].

### Analysis of multiple sequence alignment and evolutionary relationships

To support RV analysis, the sequence is encoded using amino acid residue codes and preserved as a FASTA sequence file. The acquired sequences were subsequently utilized for multiple sequence alignment (MSA) through the Clustal Omega platform (https://www.ebi.ac.uk/jdispatcher/msa/clustalo; accessed on 05 August 2025) [55]. The Mega X tool (https://www.megasoftware.net/; accessed on 05 August 2025) was employed for phylogenetic analysis on both genomes [56].

### Prediction of epitopes for MHC I, MHC II, and linear B-cell interactions

The MHC I epitopes from the G and N proteins were found using the Immune Epitope Database (IEDB) (http://tools.iedb.org/mhci/; accessed on 20 August 2025) software, with a percentile rank < 1.00 as the selection criterion [57]. This tool is a sophisticated database comprising information on MHC molecules, T-cell and B-cell epitopes, antibodies, and MHC-binding ligands identified in humans and various animal taxonomic groups, including primates and rodents [58, 59]. A cutoff percentile rank of less than 1.00 was implemented for epitope selection, which suggests that the binding affinity was improved [37]. Lower percentile rank values indicate stronger predicted peptide–MHC binding affinity [60]. The IEDB (http://tools.iedb.org/mhcii/; accessed on 20 August 2025) was applied to forecast the MHC II epitopes inside the designated areas of these proteins, and peptides with percentile ranks < 1.00 were considered potential high-affinity binders and selected for further evaluation [61]. Subsequently, the human allele reference sets were employed to predict CD8 + and CD4 + binding. The ABCpred tool (https://webs.iiitd.edu.in/raghava/abcpred/; accessed on 25 August 2025) was used to estimate the continuous B-cell epitopes of the selected portions of these proteins. This tool provides epitope predictions by analyzing the sequence features of the target antigens [58, 62]. This program uses an affinity scale method based on a Hidden Markov Model (HMM) to predict continuous B-cell epitopes [63, 64]. Additionally, the MHC-II epitopes underwent some additional analyses, including IL-10 (https://webs.iiitd.edu.in/raghava/il10pred/; accessed on 25 September 2026), IFN-gamma (https://webs.iiitd.edu.in/raghava/ifnepitope/predict.php; accessed on 25 September 2026), IL-2 (https://webs.iiitd.edu.in/raghava/il2pred/; accessed on 25 September 2026) and IL-4 (https://webs.iiitd.edu.in/raghava/il4pred/design.php; accessed on 25 September 2026) by using the respective servers. Linear B-cell epitopes were forecasted with ABCpred with a standard window length of 16 amino acids and a score cutoff of 0.51. Antigenicity was evaluated using the VaxiJen v2.0 (https://www.ddg-pharmfac.net/vaxijen/VaxiJen/VaxiJen.html; accessed on 28 August 2025) server under the viral protein model with the default threshold value of 0.4. Peptides with antigenicity scores ≥ 0.4 were considered probable antigens and retained for further analysis, whereas those scoring below this threshold were excluded. The threshold value of 0.4 was selected based on the default classification cutoff recommended for the VaxiJen viral model, which was established through performance evaluation of the prediction system [65]. AllerTOP v2.1 (threshold: 0.5) (https://www.ddg-pharmfac.net/allertop_test/; accessed on 28 August 2025) and ToxinPred (threshold: 0.0) servers (https://webs.iiitd.edu.in/raghava/toxinpred/algo.php; accessed on 28 August 2025) were deployed in order to make predictions on allergenicity and toxicity, respectively [66, 67]. The allergenicity was evaluated utilizing AllerTOP v2.1 with the server's standard ACC/k-NN algorithm configurations [66]. Also, toxicity prediction was performed using the ToxinPred server with the SVM (Swiss-Prot)-based prediction method, applying the default threshold value of 0.0 and a window length of 17 residues. Peptides were classified according to the server-generated SVM prediction score, and sequences predicted as toxic were excluded, whereas non-toxic peptides were retained for further analysis [67]. All settings were preserved according to the server’s default configuration. The selected epitopes were not chosen based only on prediction scores; additional analyses, including antigenicity, safety assessment, sequence conservation, population coverage, and MHC interaction evaluation, were also considered to improve the reliability of epitope selection.

### Evaluation of population-level coverage

The prevalence of HLA alleles varies across different geographies and ethnic communities. The population coverage of the chosen T-cell epitopes was assessed via the IEDB Population Coverage tool (http://tools.iedb.org/population/; accessed on 05 September 2025) [68, 69]. The found MHC I and MHC II epitopes, alongside their associated HLA variants, were analyzed both collectively and individually, under the default settings of the tool, predicting the widespread distribution of particular alleles across continents.

### Evaluation of epitope conservancy

Epitope conservancy was assessed using the Epitope Conservancy Analysis tool (http://tools.iedb.org/conservancy/; accessed on September 10, 2025) which determines the proportion of protein sequences that contain the epitope across different identity thresholds [70]. A sequence-based conservancy analysis was conducted to determine the proportion of PUUV protein sequences containing each selected epitope. The analysis was performed using a 100% sequence identity threshold, and the percentage of sequences matching each epitope at this identity level was calculated. Conservancy values were evaluated separately for G protein-derived and N protein-derived epitopes [70].

### Vaccine construction

The vaccine was constructed by employing specifically chosen antigenic sites from the G and N proteins. The 50S ribosomal protein L7/L12 (bl12) from *Mycobacterium tuberculosis* was used as an adjuvant, resulting in improved vaccine efficacy and immunogenicity, which subsequently enhanced the immune response, as evidenced by experimental trials. Moreover, this particular adjuvant can enhance T-cell mediated response and secretion of inflammatory cytokines [71–73]. The β-defensin-derived adjuvant bl12 was incorporated into the vaccine construct based on the reported immunomodulatory potential of defensin-derived adjuvant sequences. Such adjuvants have been associated with enhanced antigen presentation and activation of adaptive immune responses, supporting their application in multi-epitope vaccine design. Therefore, BL12 was selected as an adjuvant component to potentially enhance the immunogenicity of the designed vaccine construct [71–73]. Within the PV-GNProtecta construct, epitopes were arranged in three consecutive blocks (four MHC I, four MHC II, and four linear B-cell epitopes), joined internally by KKK linkers. This sequential organization was selected to maintain a structured separation of cytotoxic T-cell, helper T-cell, and B-cell epitope regions, thereby enabling systematic assembly of the multi-epitope vaccine construct while preserving epitope organization [74]. Linkers, including KKK and EAAAK, are designed to bind epitopes while maintaining their stability and flexibility [75, 76]. The KKK (lysine-rich) linker was used between B-cell epitopes to enhance flexibility and B-cell receptor recognition, thereby improving humoral immunogenicity [77]. The rigid EAAAK linker was used as a semi-rigid spacer to maintain structural separation between functional domains and minimize undesirable domain interactions [78]. The polyhistidine tag (6xHis tag) consists of at least six consecutive histidine residues, typically engineered at the carboxyl (C-) terminus of the protein sequence were added to the vaccine for improved stability [79]. Nonetheless, mRNA vaccines necessitate a Kozak sequence, commencing with an initial codon (AUG) at the onset of the open reading frame (ORF) and terminating with a stop codon (TGA) at the conclusion of translation [75]. Also, the construct was incorporated with the tissue plasminogen activator (tPA) leader sequence (UniProt ID: P00750) to enhance translation, along with the human β-globin (NCBI Gene ID: 3043) and α-globin (NCBI Gene ID: 3039) UTRs at the 5′ and 3′ ends, respectively, to improve mRNA stability and expression. A Pan HLA-DR-binding epitope (PADRE; AKFVAAWTLKAAA) was incorporated into the N-terminal region of the PV-GNProtecta construct, before the L7/L12 adjuvant [80]. PADRE was included to provide broad CD4⁺ T-helper cell stimulation, while L7/L12 was used as an immunostimulatory adjuvant to support innate immune activation [80, 81]. The combination of these elements was designed to improve antigen-specific immune responses and enhance the immunogenic potential of the vaccine construct. Finally, the vaccine candidate was designated as PV-GNProtecta to signify its design elements. The PV-GNProtecta refers to Puumala Virus-Glycoprotein and Nucleoprotein Protection, meaning a protective vaccine focusing on G and N proteins of the Puumala virus.

### Assessment of biophysical characteristics

The vaccine’s biophysical characteristics were analyzed through the ExPASy ProtParam web tool (http://web.expasy.org/protparam/; accessed on 18 September 2025), which provides information regarding total atom count, molecular weight, cumulative amino acid quantity, instability parameter, aliphatic index, grand mean of hydropathicity (GRAVY), and isoelectric point (pI) using the default parameters [82]. Furthermore, the solvent affinity of the vaccine was assessed utilizing the SOSUI (https://harrier.nagahama-i-bio.ac.jp/sosui/mobile/; accessed on 18 September 2025) and SOLpro (https://scratch.proteomics.ics.uci.edu; accessed on) servers [83–86]. Subsequently, the allergenicity of the vaccine was assessed through the AllerTOP v.2.1 (https://www.ddg-pharmfac.net/allertop_test/; accessed on 18 September 2025) tools [66]. The VaxiJen v2.0 (https://www.ddg-pharmfac.net/vaxijen/VaxiJen/VaxiJen.html; accessed on 18 September 2025) server predicted the antigenicity of the vaccine [65, 87]. PV-GNProtecta vaccine construct was analyzed for potential similarity with human proteins using BLASTp (https://blast.ncbi.nlm.nih.gov/Blast.cgi?PAGE=Proteins; accessed on 16 September 2026) against the Homo sapiens RefSeq protein database [88]. The analysis was performed to identify possible sequence homology that may contribute to unintended immune cross-reactivity.

### Modeling of secondary structure

Secondary structure prediction and modeling for the designed vaccine was carried out using three online servers—GOR4 (https://npsa.lyon.inserm.fr/cgi-bin/npsa_automat.pl?page=/NPSA/npsa_gor4.html; accessed on 25 September 2025) [89], SOPMA (accessed on 25 September 2025)[90], and PSIPRED (https://bioinf.cs.ucl.ac.uk/psipred/; accessed on 25 September 2025) [91], with all parameters applied under default configurations [92, 93]. Alternatively, GOR4 uses evolutionary data, information theory, and Bayesian statistics to estimate secondary arrangement, whereas SOPMA uses a neural network with a 73.5% and 69.5% accuracy rate, respectively [90, 94]. Additionally, PSIPRED utilises deep learning and PSI-BLAST to predict protein secondary structural elements [95].

### Prediction, refinement, and validation of tertiary structure

The three-dimensional (3D) conformation of the vaccine candidate was predicted with AlphaFold 3 (https://www.deepmind.com/research/highlighted-research/alphafold; accessed on 10 October 2025), a deep learning neural network that was trained on extensive protein sequence and structure databases. This approach enables the precise modeling of protein folding and conformational states [96, 97]. Subsequently, the GalaxyWEB tool (https://galaxy.seoklab.org/cgi-bin/submit.cgi?type=REFINE; accessed on 21 October 2025) was implemented to optimize the vaccine construction [98]. Structural validation was then performed using the QMEAN server of SWISS-MODEL web (https://swissmodel.expasy.org/qmean/; accessed on 26 October 2025) and the SAVES v6.1 (https://saves.mbi.ucla.edu/; accessed on 26 October 2025) suite for QMEAN *Z*-score and ERRAT analyses, respectively, while PDBsum (https://www.ebi.ac.uk/thornton-srv/databases/pdbsum/; accessed on 29 October 2025) provided PROCHECK-based Ramachandran plots for final model assessment, with all analyses performed using the respective servers' default parameters [99–101].

### Disulfide bond analysis

To identify potential structural stabilization sites, disulfide engineering analysis was performed using the Disulfide by Design 2 (v2.13) server (http://cptweb.cpt.wayne.edu/DbD2/; accessed on 15 October 2025) [102, 103]. The server was used to identify favorable residue pairs suitable for cysteine substitution and potential disulfide bridge formation. The prediction was based on geometric parameters associated with stable disulfide formation, including Cα-Cα distance, Cβ-Cβ distance, and *χ*3 dihedral angle. The identified residue pairs were considered potential candidates for disulfide engineering to potentially improve the conformational stability of the vaccine construct.

### Evaluation of conformational B-cell epitope

The discontinuous B-cell epitope regions within PV-GNProtecta were found via the ElliPro service (http://tools.iedb.org/ellipro/; accessed on 3 November 2025) by uploading the improved tertiary structure [104]. Discontinuous B-cell epitopes were forecasted utilizing the default settings of the ElliPro server, applying a minimum Protrusion Index (PI) score of 0.5 and a maximum residue-to-residue distance of 6 Å to the tertiary structure of PV-GNProtecta [104]. The ElliPro prediction tool detects discontinuous B-cell epitopes by mapping amino acid clusters within surface-exposed regions that represent the native antigen conformation and the possibility for antibody interaction [105].

### Docking of vaccine construct with TLRs

Toll-like receptors (TLRs), particularly human TLR4, serve a key function in identifying specific pathogen-associated molecular patterns (PAMPs), thereby initiating comprehensive immune responses [106, 107]. Their capacity to provoke strong immunological responses renders them adaptable and essential targets for augmenting vaccine-induced immunity [108, 109]. Thus, the 3D structural coordinates of human TLR4 (PDB: 3FXI) were acquired from the Protein Data Bank (www.rcsb.org; accessed on 7 November 2025) for conducting computational docking with the vaccine construct and the specified TLRs [106, 110, 111]. Prior to molecular docking, the TLR4 receptor structure was prepared by selecting only chain A, while removing co-crystallized ligands, water molecules, and non-protein heteroatoms. Hydrogen atoms were subsequently added, and protonation states were assigned to optimize the receptor structure for docking analysis. These steps were conducted using PyMOL (https://www.pymol.org/; accessed on 20 November 2025). Subsequently, molecular docking was carried out using the ClusPro 2.0 server (https://cluspro.bu.edu/; accessed on 12 November 2025) to evaluate the molecular interactions between the designed vaccine construct and human TLR4 (PDB ID: 3FXI), thereby assessing the predicted molecular interaction between the PV-GNProtecta construct and the human TLR4 receptor [112]. This server performed docking using the default docking parameters. The antibody mode option was not activated because the docking partners consisted of the TLR4 receptor and the PV-GNProtecta vaccine construct rather than an antibody–antigen complex requiring complementarity-determining region (CDR) masking. Afterwards, the ligand-receptor complex was visualized using PyMOL (https://www.pymol.org/) and analyzed using the PDBsum servers (https://www.ebi.ac.uk/thornton-srv/databases/pdbsum/; accessed on 20 November 2025) [113].

### Docking analysis between epitopes and MHC proteins

The HDOCK online tool (http://hdock.phys.hust.edu.cn/; accessed on 28 November 2025) was employed to facilitate the molecular docking analysis [114]. The docking of CD8⁺ and CD4⁺ epitopes with their corresponding MHC I (PDB id: 2XPG) and MHC II (PDB id: 1DLH) molecules was performed using the HDOCK server under default global docking conditions. No binding-site residues were specified for either the receptor or ligand, allowing unbiased docking without predefined interaction restraints. The HDOCK algorithm performs protein–protein docking through FFT-based rigid-body sampling followed by knowledge-based scoring using the ITScorePP scoring function [114]. This method facilitated the assessment of the binding affinity together with the positioning of the engineered epitopes inside the MHC peptide-binding groove, thus prioritizing the support of selecting epitopes.

### Evaluation of binding affinity of the vaccine-receptor complex

The MM-GBSA (Molecular Mechanics–Generalized Born Surface Area) method was employed via the HawkDock web platform (https://cadd.zju.edu.cn/hawkdock/; accessed on 06 December 2025) to assess the binding free energies of PV-GNProtecta-TLR4, achieving accuracies ranging from 80 to 95% across both crystal-derived and predicted protein structures [115–117]. Although MM-PBSA is widely used for binding free energy estimation, MM-GBSA was selected in this study due to its efficiency and reliability in evaluating relative binding energetics of protein–protein complex [118]. This methodology assesses various characteristics, particularly interactions including binding and electrostatic forces, polar and nonpolar contributions, van der Waals contacts, and the implications of complex formation [37]. Protein–protein binding affinity of the PV-GNProtecta-TLR4 complex was further estimated using the PRODIGY web server (https://haddock.chem.uu.nl/prodigy; accessed on 14 September, 2026) [119]. The predicted binding free energy (ΔG) and dissociation constant (Kd) were obtained at 25 °C to quantitatively evaluate the strength of the vaccine–receptor interaction.

### Molecular dynamics (MD) simulation analysis

MD simulation was utilized to evaluate the structural stability and integrity of the PV-GNProtecta-TLR4 complex by analyzing Root Mean Square Deviation (RMSD), Root Mean Square Fluctuation (RMSF), Radius of Gyration (Rg), and intermolecular interactions [120]. MD simulations of the PV-GNProtecta-TLR4 complex were performed in this investigation over a duration of 100 ns, utilizing the Desmond module from Schrödinger (Release 2023-1) within a Linux environment. The original PV-GNProtecta complex for the MD simulation was acquired via docking studies. The PV-GNProtecta-TLR4 complex was preprocessed using the Protein Preparation Wizard of Maestro v13.5. Missing hydrogen atoms were added, and protonation states of ionizable residues were assigned at pH 7.4 using Epik. The prepared complex was subsequently optimized and minimized using the OPLS4 force field.

The docked complex was solvated using the Transferable Intermolecular Interaction Potential 3 Points (TIP3P) water model in an orthorhombic periodic boundary system with a 10 Å buffer distance. The system was neutralized using Na+ counterions, and 0.15 M NaCl was added to maintain physiological ionic strength.

Before the production simulation, the solvated system was subjected to the standard Desmond relaxation protocol. The relaxation consisted of a 100 ps Brownian dynamics NVT stage at 10 K with restraints on solute heavy atoms (50.0 kcal mol⁻^1^ Å⁻^2^), followed by a 12 ps NVT stage at 10 K using a Langevin thermostat with a relaxation time of 0.1 ps. Subsequently, the system underwent sequential NPT relaxation stages consisting of two restrained NPT stages (12 ps each) and a final 24 ps unrestrained NPT stage. The NPT stages employed Langevin thermostat and barostat coupling with relaxation times of 0.1 ps and 50 ps, respectively, for restrained stages and 2.0 ps for the final unrestrained stage.

The production molecular dynamics simulation was performed for 100 ns under the NPT ensemble at 300 K and 1.01325 bar pressure. A base integration timestep of 0.002 ps (2 fs) was applied, with multiple-time-step integration settings of 0.002, 0.002, and 0.006 ps. The trajectory was recorded at 100 ps intervals. Long-range electrostatic interactions were treated using the Useries method with a nonbonded cutoff radius of 9.0 Å and an Ewald tolerance of 1 × 10⁻^1^⁰. Periodic boundary conditions were applied, and no positional restraints were used during the production simulation.

The MD trajectory of the complete PV-GNProtecta-TLR4 assembly was analyzed as a single interacting system, and RMSD, RMSF, radius of gyration, hydrogen bonding, PCA, and DCCM analyses were performed to evaluate the overall conformational behavior and interaction dynamics of the vaccine-receptor complex after docking. PCA and DCCM analyses were conducted using the Bio3D package in R 4.3.1, while post-simulation binding free energy calculations were performed using Prime MM-GBSA v3.0.

#### Post-simulation MM-GBSA analysis

Post-simulation MM-GBSA analysis was performed using the Prime MM-GBSA module (Schrödinger Maestro v13.5) to evaluate the change in binding energetics of the PV-GNProtecta-TLR4 complex following the 100 ns molecular dynamics simulation. The calculation was performed using the default Prime MM-GBSA parameters. For comparative endpoint analysis, the initial (0 ns) and final (100 ns) trajectory structures were extracted from the MD simulation and analyzed to estimate the binding free energy (ΔG Bind) and individual energy components, including van der Waals, Coulombic, hydrogen bonding, lipophilic, packing, and solvation contributions.

### Optimization of codon and in silico cloning

The designed vaccine sequence underwent codon optimization performed using the Java Codon Adaptation Tool (JCat) server (http://www.jcat.de/Start.jsp; accessed on 03 January 2026) [121], selecting *Escherichia coli* strain K12 as the preferred expression host. *E. coli* K-12 was selected as the reference host for JCat codon optimization due to its well-characterized codon usage profile and established use in recombinant protein expression systems. This computational optimization was performed to improve codon compatibility of the vaccine construct and facilitate future experimental validation. The codon-refined gene sequence was inserted into the *E. coli* pET-28a (+) plasmid vector via the Eco53kI and EcoRV restriction sites, adhering to the conventional cloning process. Additionally, the cloning design was computationally performed through the SnapGene (https://www.snapgene.com/) tool [58].

### Computational analysis of immune response

The C-ImmSim web server (https://kraken.iac.rm.cnr.it/C-IMMSIM/index.php; accessed on 02 February 2026) was used to mimic the immunological reaction generated by the designed PV-GNProtecta vaccine candidate [122]. It forecasts antibody-mediated and cellular immune reactions using agent-based modeling. A three-dose vaccination protocol was employed to assess vaccine-induced immunogenicity, with doses administered four weeks apart. The vaccine was administered at simulation time steps 1, 28, and 178 in the C-ImmSim analysis in order to simulate a prime-boost immunization approach and assess the primary and subsequent immune responses. The basic immune reaction was started by the first treatment, and the establishment of immunological memory, secondary immune responses, and immune boosting were evaluated by the successive administrations. A simulation volume of 50 and a step size of 1000 were employed for the analysis, while all other parameters were preserved at the server’s default settings.

### Structural modeling of the mRNA vaccine construct

The codon-adapted DNA sequence corresponding to the PV-GNProtecta vaccine candidate, generated through JCat, was subsequently transformed into its matching RNA sequence using the Biomodel web server (https://biomodel.uah.es/en/lab/cybertory/analysis/trans.htm; accessed on 08 February 2026) [123]. Following this, the secondary structural profile of the mRNA vaccine was ascertained using the RNAfold web program (https://rna.tbi.univie.ac.at/cgi-bin/RNAWebSuite/RNAfold.cgi; accessed on 08 February 2026), which evaluates the energetic profiles of the specified mRNA conformation based on calorimetric principles, with the default parameters of both web servers applied [124–126].

## Result

### Protein sequence acquisition

**Fig. 1:**
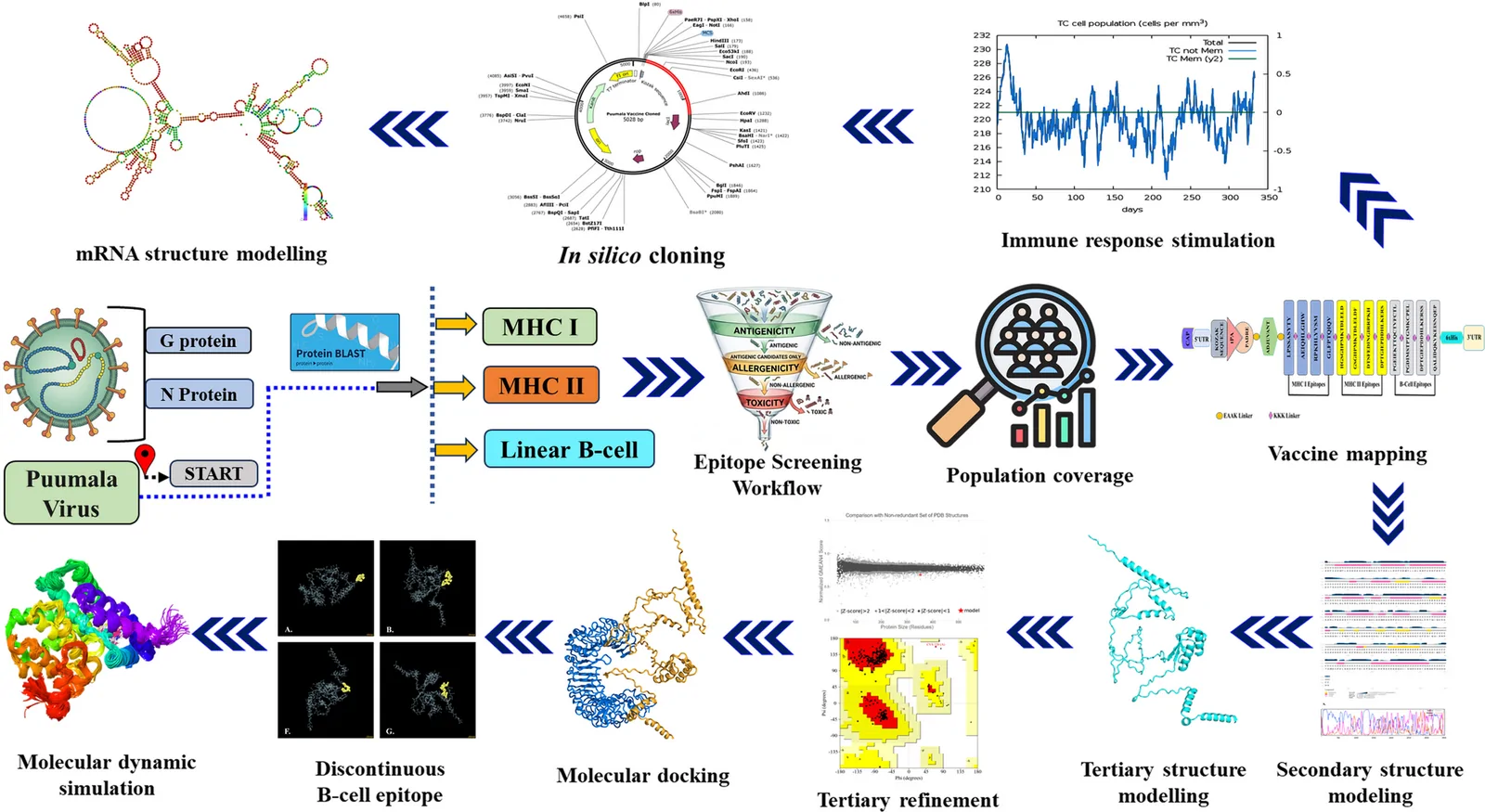
A framework of the immunoinformatics-driven workflow employed for the design and assessment of a multiepitope mRNA vaccine candidate targeting PUUV. (The workflow schematic was designed using Microsoft PowerPoint)

The amino acid sequences of proteins G and N were retrieved from UniProt and were used for further steps of the study. The study design is schematically depicted in Fig. 1.

### Physicochemical and structural characterization of selected proteins

Before epitope screening, the selected G and N proteins were further characterized for signal peptides, transmembrane regions, and subcellular localization (Supplementary Table S1). SignalP analysis identified an N-terminal signal peptide in the G protein with cleavage predicted between residues 24 and 25, whereas no signal peptide was detected in the N protein. DeepTMHMM analysis predicted three transmembrane regions in the G protein and one potential transmembrane region in the N protein. UniProt annotation showed that G protein is associated with virion and host membrane compartments, while N protein is mainly associated with the virion and intracellular regions.

### Analysis of multiple sequence alignment and evolutionary relationships

**Fig. 2:**
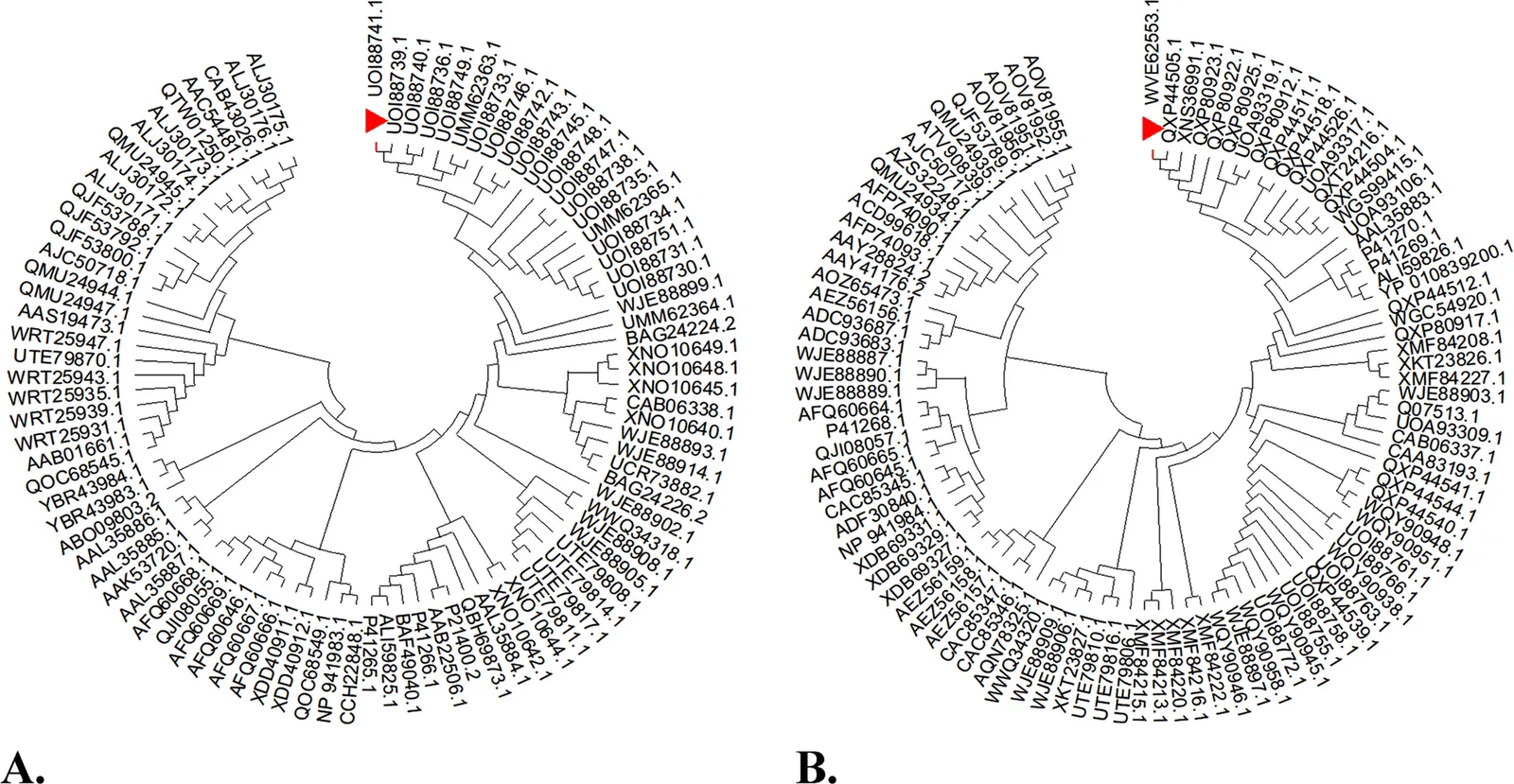
Phylogenetic screening of target PUUV antigens. **A** Evolutionary clustering of G protein variants. **B** Evolutionary clustering of N protein lineages

Sequence homology of the G and N proteins was searched by BLAST at NCBI using the BLASTp method. Multiple protein sequences were obtained from the NCBI protein database based on sequence availability and relevance to PUUV G and N proteins. Subsequently, 93 and 100 protein sequences for the G and N proteins, respectively, were collected from different strains, originating from various regions around the world and Clustal Omega was employed to conduct multiple sequence alignment (MSA). Further, phylogenetic trees were prepared by MEGA X, separately for the G protein (Fig. 2A) and N protein (Fig. 2B).

### Prediction of epitopes for MHC I, MHC II, and linear B-cell interactions

A set of 12 epitopes, consisting of 4 MHC I, 4 MHC II, and 4 linear B-cell epitopes were chosen from the obtained proteins. These epitopes were prioritized for the resultant vaccine formulation because they were predicted to have high antigenicity along with non-allergenic, non-toxic properties in MHC I, MHC II, and linear B-cell predictions (Table 1 and Supplementary Table S2).

**Table 1:** List of prioritized MHC I, MHC II, and linear B-cell epitopes with their antigenicity, allergenicity, toxicity, Il-10, IFN gamma, IL-2, and IL-4 predictions

Protein	Epitope	Peptide	Relative rank	Antigenicity	Allergenicity	Toxicity	IL-10	IFN gamma	IL-2	IL-4
G	MHC I	LPSSASYTY	0.01	0.4905 (Yes)	No	No				
		AEIQHLGHW	0.01	0.8097 (Yes)	No	No				
	MHC II	HGSGIIPMKTDLELD	0.14	0.7868 (Yes)	No	No	No	Yes	Yes	Yes
		GSGIIPMKTDLELDF	0.14	1.2985 (Yes)	No	No	No	Yes	No	Yes
	B-cell	PGEIEKTTQCTVFCTL	0.94	0.8609 (Yes)	No	No				
		PGDIMSTPTGMKCPEL	0.93	0.4989 (Yes)	No	No				
N	MHC I	RPKHLYVSM	0.01	1.2128 (Yes)	No	No				
		GLFPTQIQV	0.01	0.9432 (Yes)	No	No				
	MHC II	DTSFEDINGIRRPKH	0.01	0.4157 (Yes)	No	No	No	Yes	No	No
		DPTGIEPDDHLKERS	0.07	1.0369 (Yes)	No	No		Yes		
	B-cell	DPTGIEPDDHLKERSS	0.94	0.9441 (Yes)	No	No				
		QALIDQKVKEISNQEP	0.92	0.7490 (Yes)	No	No				

### Evaluation of population-level coverage

**Fig. 3:**
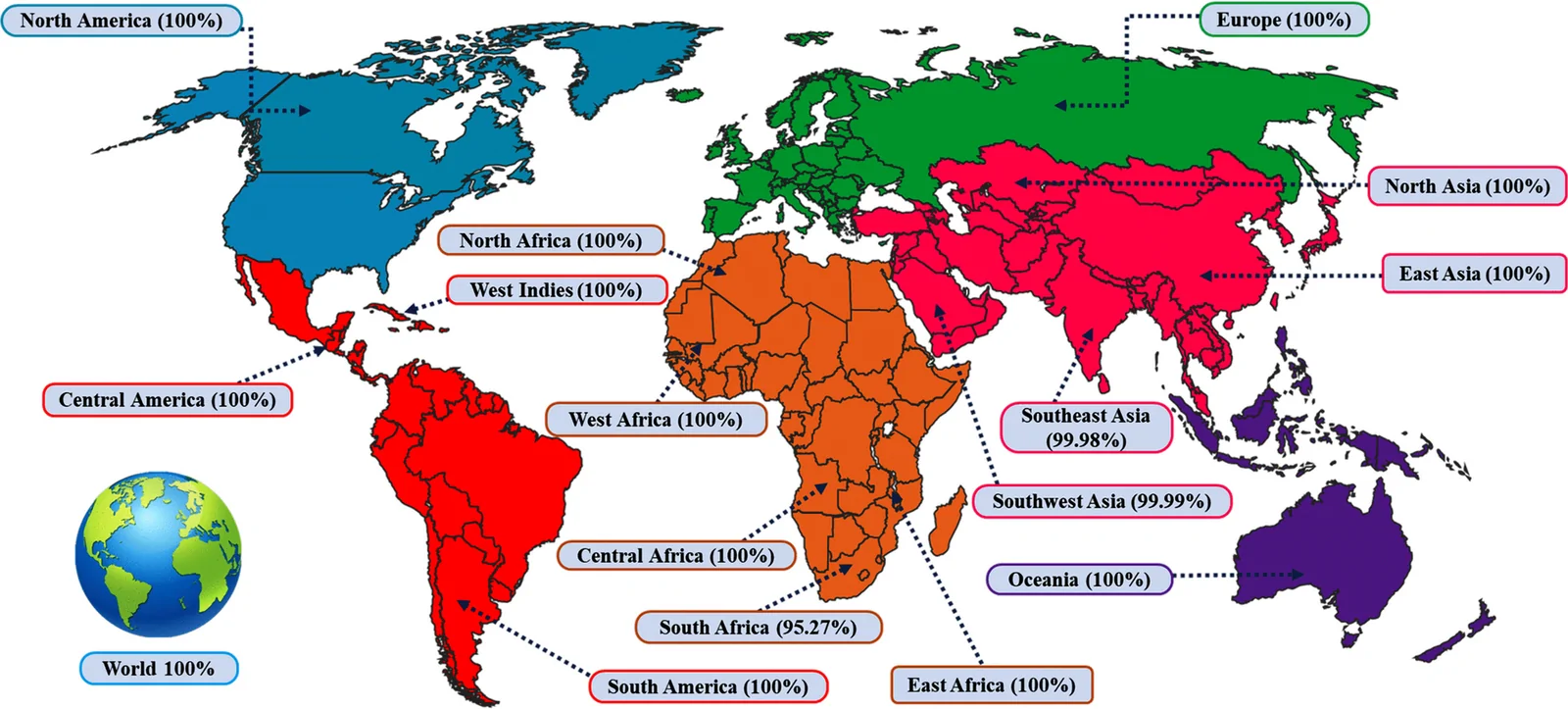
Geographical mapping of predicted population coverage. Visual representation of the cumulative T-cell epitope coverage across major global regions. Individual labels detail the specific percentage of population coverage achieved within each distinct zone, concluding with an overall global coverage estimation. (The geographic population-coverage map was generated using Microsoft PowerPoint, based on the percentage output of the IEDB Population Coverage tool)

The coverage of the population was predicted at or nearly 100% worldwide (Fig. 3). Especially, Oceania, Central America, South America, Europe, East Africa, West Africa, Central Africa, North Africa, West Indies, Northeast Asia, South Asia, North America, Central America, West Africa, and East Asia predicted the highest coverage, at 100%, while South Africa had the lowest coverage, at 95.27% (Supplementary Fig. S1).

### Evaluation of epitope conservancy

Evaluation of sequence conservation revealed variable levels of conservancy among the selected epitopes derived from the G and N proteins. The G protein-derived epitopes exhibited conservancy values ranging from 38.71 to 100.00%, whereas N protein-derived epitopes showed conservancy values ranging from 51.00 to 100.00% (Supplementary Table S3). Therefore, these findings indicate that the selected epitopes exhibit heterogeneous sequence conservation patterns among the analyzed PUUV variants.

### Vaccine construction

**Fig. 4:**
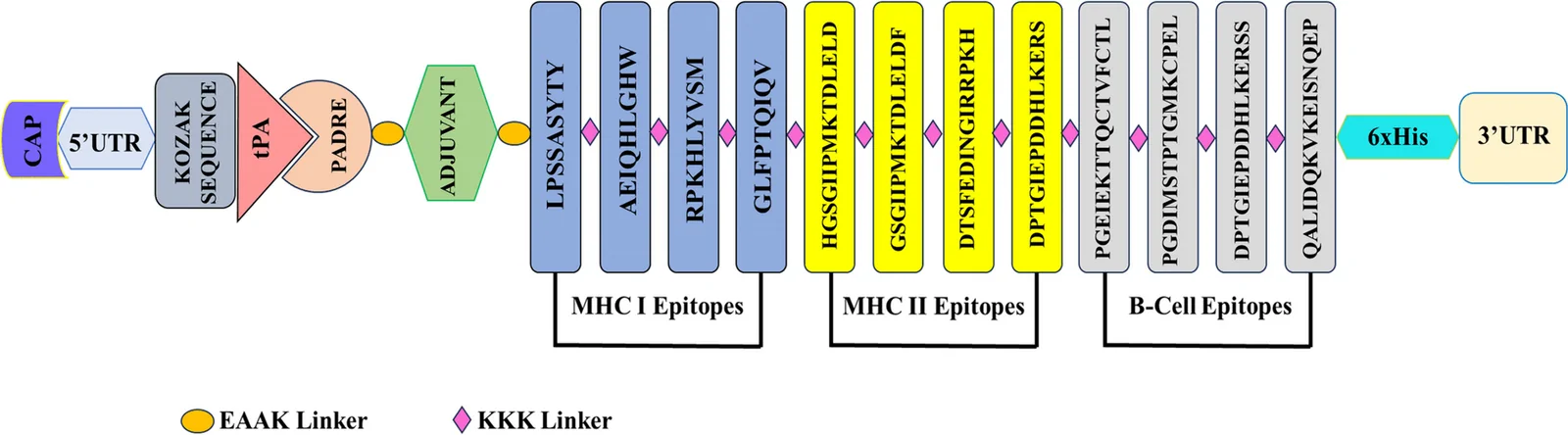
Schematic design and primary sequence of the engineered PV-GNProtecta vaccine construct. (The schematic vaccine construct diagram was designed using Microsoft PowerPoint)

The selected epitopes derived from G and N were assembled to construct the PV-GNProtecta (Fig. 4). In this approach, the EAAAK rigid linker was introduced to efficiently integrate the adjuvants along with selected epitopes in the vaccine construct, while KKK linkers were employed to conjugate the MHC I, MHC II, and linear B-cell epitopes. Also, a 6xHis tag, a Kozak sequence, tPA, and UTRs at the 5′ and 3′ ends were incorporated within the peptide sequence.

### Assessment of biophysical characteristics

Detailed computational analysis via the ExPASy ProtParam server characterized the PV-GNProtecta as a 352-polypeptide chain with a molecular mass of 38.60 kDa. The protein exhibited a slightly basic nature (pI = 9.31), driven by a surplus of positively charged residues (68) relative to negatively charged ones (54). Also, stability assessments depicted an instability index of 27.30 and an aliphatic index of 77.73, suggesting a robust and thermostable structure. The PV-GNProtecta suggested a strongly hydrophilic profile (GRAVY = − 0.613) and a high predicted solubility index (0.954) for *E. coli* expression systems. Crucially, the vaccine was projected to be highly antigenic, non-allergenic, and non-toxic, satisfying the primary safety requirements for a viable immunogen (Table 2). BLASTp analysis against the human RefSeq protein database also showed no significant similarity between PV-GNProtecta and human proteins.

**Table 2:** In silico biophysical evaluation, primary structural metrics, and safety profiles of the proposed PV-GNProtecta vaccine

Biophysical features	Scores
Amino acid number	352
pI	9.31
Molecular weight (kDa)	38.60
Total anionic residues (aspartate + glutamate)	54
Total cationic residues (arginine + lysine)	68
Total atomic count	5540
The predicted half-life	● 4.4 h (Reticulocytes of mammalian origin, in vitro) ● > 20 h (yeast, in vivo) ● > 10 h (*E. coli*, in vivo)
The instability index (II)	27.30 (Stable)
Aliphatic index	77.73
Grand average of hydropathicity (GRAVY)	− 0.613
Soluble nature/Scratch	Solubility upon overexpression (0.954485)
Allergenic response/AllerTOP v. 2.1	Likely non-allergen
Immunogenicity/Vaxijen	0.6699 (Expected ANTIGEN)
Predicted scaled solubility	0.754

### Modeling of secondary structure

The secondary structure of the PV-GNProtecta was inferred using the GOR4, SOPMA, and PSIPRED tools. The GOR4 server predicted that the PV-GNProtecta is composed of approximately 53.41% (188) alpha helices, 11.93% (42) extended beta-strands, and 34.66% (122) random coils (Supplementary Fig. S2). In addition, SOPMA analysis estimated the secondary structure to be composed of 44.32% (156) alpha helices, 42.90% (151) random coils, 8.24% (29) extended strands, and 4.55% (16) beta-turns (Supplementary Fig. S2). PSIPRED was employed to result in a three-state prediction of the secondary conformation of the PV-GNProtecta, comprising helices, coils, and strands (Supplementary Fig. S2). The predicted secondary structure showed a combination of ordered and flexible regions, with alpha helices and beta-strands representing the major structural elements. The comparable patterns observed among GOR4, SOPMA, and PSIPRED support the consistency of the prediction (Supplementary Table S4).

### Prediction, refinement, and validation of tertiary structure

The AlphaFold 3 server generated five alternative structural models of PV-GNProtecta. Among those, the model (Supplementary Fig. S4A) with the QMEAN *Z*-score of − 6.85 from the SWISS-MODEL server (Supplementary Fig. S4B) was chosen for detailed analysis. The GalaxyWeb server then produced a refined 3D model (Supplementary Fig. S5C) with an RMSD of 0.989, a MolProbity score lowered to 1.149 from 2.682 in the unrefined structure, and a Ramachandran favored region of 98.6%, improved from 86.0% in the unminimized model. In addition, the refined configuration obtained a QMEAN *Z*-score of − 2.71 (Supplementary Fig. S4D) and an ERRAT value of 91.4 (Supplementary Fig. S3), both of which are within the acceptable range for proteins of equivalent size. Out of 352 residues, Ramachandran analysis via PDBsum revealed that 95.5% were located in the most preferred regions, 4.1% in additional allowed zones, 0.0% in marginally allowed zones, and 0.3% in the excluded zones (Supplementary Fig. S3).

### Disulfide bond analysis

Although nine residue pairs were identified by Disulfide by Design 2 as potential candidates, four pairs were prioritized based on favorable geometric and energetic parameters, including PHE_81-ALA_131, LYS_219-GLY_220, GLY_89-GLY_142, and GLU_80-ALA_150, with the energy of 0.94, 2.71, 4.19, and 4.34 kcal/mol, respectively and Chi3 value of + 99.74, + 100.12, + 75.01, and − 85.82, sequentially (Supplementary TableS5). These pairs represent predicted locations for possible cysteine substitution and engineered disulfide bond formation rather than native disulfide linkages.

### Evaluation of conformational B-cell epitope

Altogether 167 conformational B-cell epitope residues from the 352 amino acids were obtained using the Discotope 2.0 tool for the PV-GNProtecta. The ElliPro server detected 13 discontinuous B-cell epitopes, which exhibited different scores within the range of 0.526 to 0.967, each with a distinct residue count (Supplementary Table S6).

### Docking of vaccine c onstruct with TLRs

**Fig. 5:**
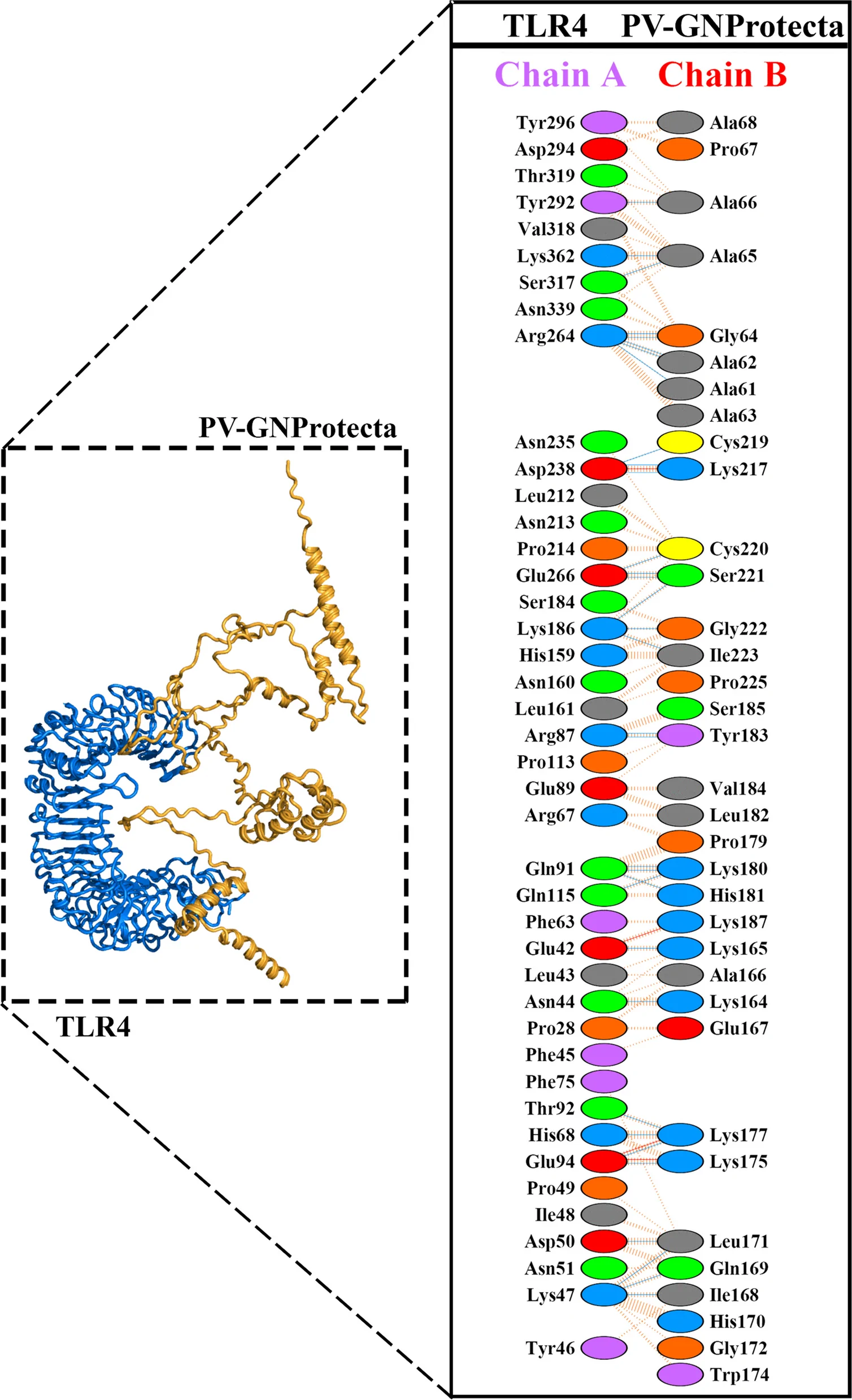
The predicted interaction pattern between PV-GNProtecta and the human TLR4 receptor obtained through molecular docking analysis. TLR4 represents the host immune receptor, while PV-GNProtecta represents the designed vaccine construct interacting with the receptor. Residues involved in intermolecular contacts between PV-GNProtecta and TLR4 are displayed, highlighting the potential binding interface and stability of the complex. Different colored residues represent interacting amino acids from both chains contributing to the formation of HBs, salt bridges, and non-bonded interactions within the PV-GNProtecta-TLR4 complex

ClusPro 2.0 server was employed to perform docking analysis between the human TLR4 receptor and PV-GNProtecta. Among the provided docked structures, the structure with the central and lowest energy score of − 1060.6 kJ/mol was selected for subsequent structural and dynamic analyses (Supplementary Table S7). Moreover, 263 non-bonded contacts, 33 hydrogen bonds (HBs), and 4 salt bridges were found within the PV-GNProtecta–TLR4 analyzed from PDBsum (Fig. 5 and Table 3).

**Table 3:** Molecular docking interaction profile of the PV-GNProtecta vaccine construct with the human TLR4 receptor, showing the calculated binding energies and major intermolecular interactions

Ligand-receptor	Weighted score		Numbers of Interactions			
	Lowest energy (KJ/mol)	Center (KJ/mol)	Salt bridges	Disulfide bonds	Hydrogen bonds	Non-bonded contacts
PV-GNProtecta-TLR4	− 1060.6	− 1060.6	4	–-	33	263

### Docking analysis of epitopes with MHC proteins

**Fig. 6:**
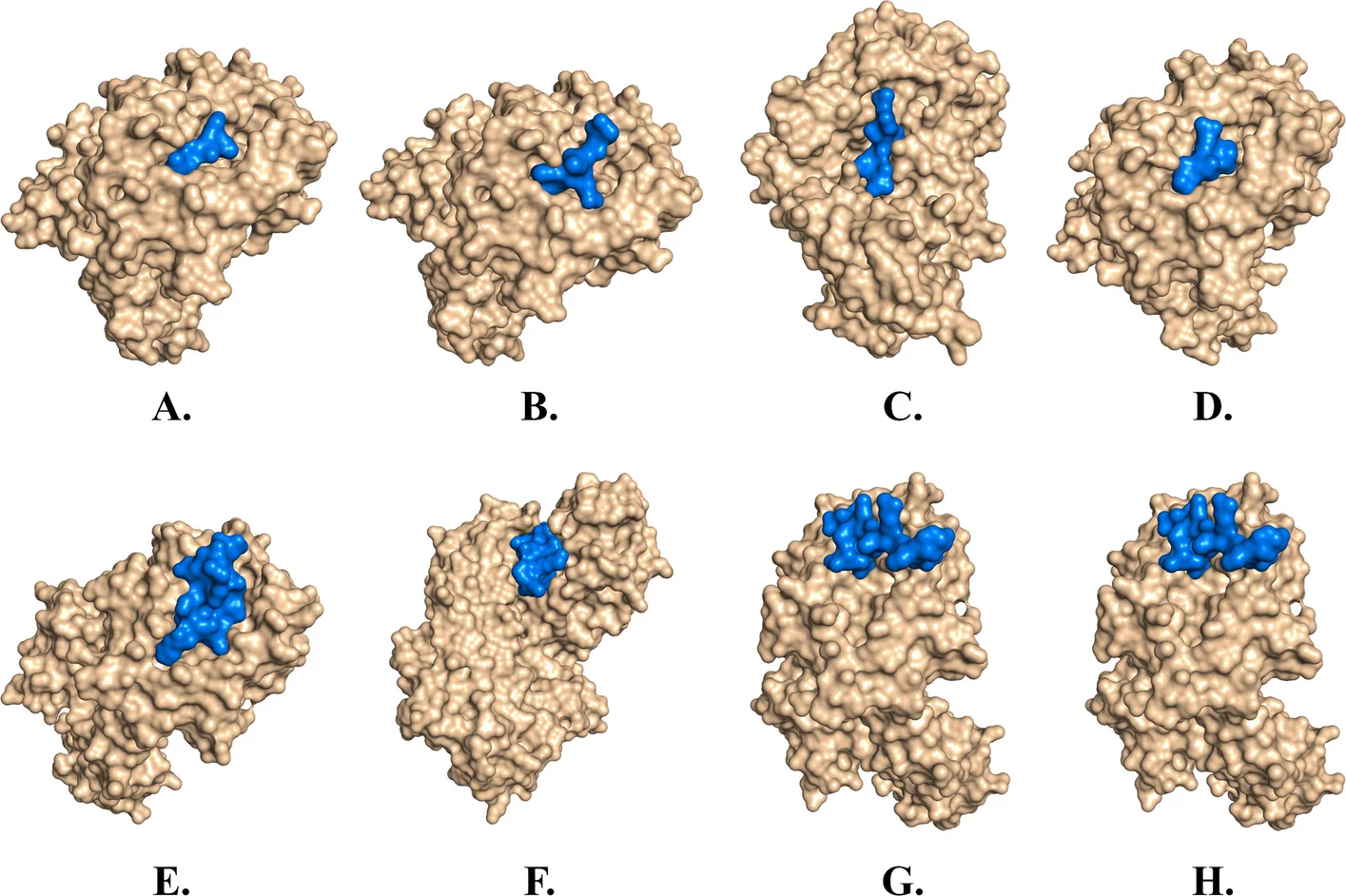
Docking conformations of the selected vaccine epitopes with human MHC molecules. **A**–**D** present the binding patterns of the identified CD8+ T-cell epitopes with MHC I proteins, while **E**–**H** show the interactions of predicted CD4+ T-cell MHC peptide candidates bound to MHC II proteins. In all panels, the epitopes are displayed in blue within the binding pockets of the respective MHC molecules

The HDOCK server was applied to dock selected CD8⁺ and CD4⁺ epitopes with human MHC I and MHC II molecules. For MHC I docking with CD8⁺ epitopes, corresponding binding scores for LPSSASYTY (Fig. 6A), AEIQHLGHW (Fig. 6B), RPKHLYVSM (Fig. 6C), and GLFPTQIQV (Fig. 6D) were − 146.24, − 163.98, − 196.47, and − 187.17 kJ/mol, respectively. While for MHC II docking with CD4⁺ epitopes, the scores were − 197.86, − 173.75, − 190.04, and − 152.50 kJ/mol for HGSGIIPMKTDLELD (Fig. 6E), GSGIIPMKTDLELDF (Fig. 6F), DTSFEDINGIRRPKH (Fig. 6G), and DPTGIEPDDHLKERS (Fig. 6H), sequentially.

### Evaluation of binding affinity of the vaccine-receptor complex

The HawkDock server was applied to evaluate the interaction energetics between PV-GNProtecta and the TLR4 complex. The analysis revealed an overall binding energy of − 166.99 kcal/mol (Table 4), indicating a stable association between the PV-GNProtecta and TLR4. The calculated energy components, including electrostatic interaction energy (ELE), van der Waals force contribution (vdW), generalized born solvation component (GB), and surface area-related energy (SA), were − 2571.51, − 182.56, 2614.56, and − 27.48 kcal/mol, respectively (Table 4). To further quantify the interaction between PV-GNProtecta and TLR4, PRODIGY analysis was performed. The complex showed a predicted binding free energy (ΔG) of − 17.6 kcal/mol and a dissociation constant (Kd) of 1.3 × 10^–13^ M at 25 °C (Supplementary Table S8).

**Table 4:** Binding energetics of the PV-GNProtecta-TLR4 complex estimated using MM-GBSA

Complex name	vdW	ELE	GB	SA	ΔG Bind
PV-GNProtecta-TLR4	− 182.56	− 2571.51	2614.56	− 27.48	− 166.99

### MD simulation

To gain a deeper view of the dynamic characteristics of the PV-GNProtecta-TLR4 molecular assembly, a series of computational analyses was performed, including RMSD, RMSF, Rg, and HB profiling. These evaluations provided a comprehensive understanding of the conformational stability and dynamic flexibility of the PV-GNProtecta-TLR4 system during the simulation period. In addition, binding affinity was estimated through the MM-GBSA framework, implemented via the Prime module in Schrödinger Maestro with default parameters. Principal component assessments (PCA) and dynamic cross-correlation matrix (DCCM) analyses were applied to the simulation trajectory to characterize system motions and the distribution of energy. These analyses were carried out using the Bio3D package in R, with customized R scripts employed in case of PCA and DCCM computations.

#### RMSD assessment

**Fig. 7:**
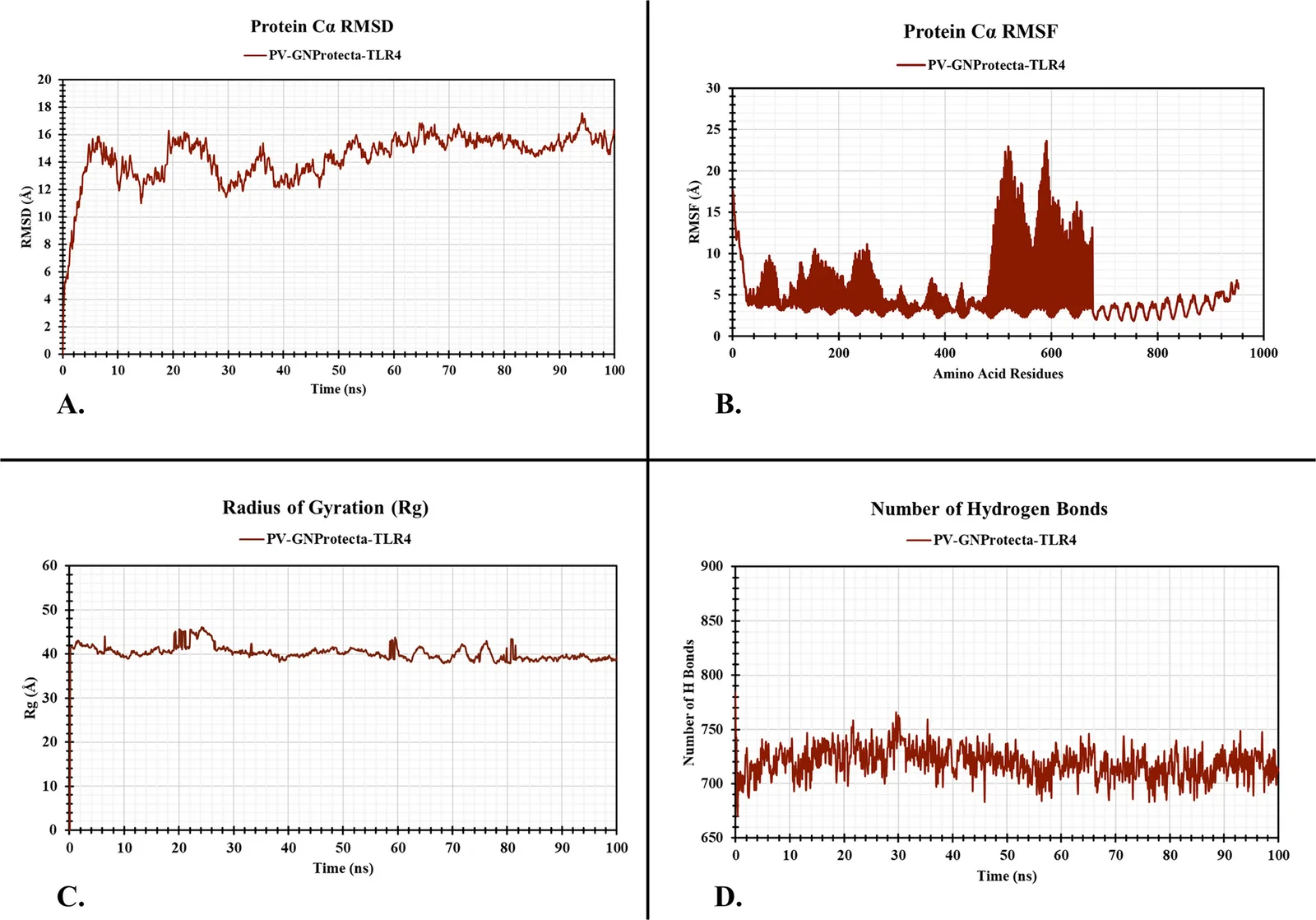
MD simulation results of the PV-GNProtecta-TLR4 complex showing **A** RMSD analysis, **B** RMSF profile, **C** Rg, and **D** Number of HB interactions throughout the simulation period

The RMSD of the PV-GNProtecta-TLR4 complex was computed to evaluate system stability, with conformational changes in the protein reflected by variations in RMSD values [127]. The mean RMSD for the PV-GNProtecta-TLR4 molecular assembly was 14.37 Å. The RMSD trajectory of the PV-GNProtecta-TLR4 assembly suggested an initial rapid increase during the early phase of the simulation, reaching approximately 15 Å within the first 10 ns (Fig. 7A and Supplementary Table S9). Following this period, the RMSD values fluctuated within a relatively consistent range of ~12–17 Å throughout the remaining simulation time, suggesting that the PV-GNProtecta-TLR4 complex underwent dynamic equilibration and remained within a confined range of conformational fluctuations after the initial structural adjustment. Additionally, minor fluctuations were observed after 50 ns (Fig. 7A and Supplementary Table S9), which may correspond to localized conformational rearrangements within the complex during the dynamic simulation process.

#### RMSF assessment

RMSF analysis was carried out to investigate the stability and dynamic movement of individual amino acid residues within the complex, thereby providing insight into its dynamic structural behavior [128]. The RMSF profile showed an average value of 5.48 Å for the PV-GNProtecta-TLR4 complex. The minimum and maximum fluctuations of 1.867 Å and 23.625 Å, respectively, were observed at residues GLN_430 and LYS_309 (Fig. 7B and Supplementary Table S10). During the 100 ns simulation, the complex exhibited significant fluctuations in the protein peak regions, notably at residues GLU_48, GLN_78, LYS_91, ALA_140, GLY_172, LYS_201, ASP_229, LYS_273, LYS_309, VAL_338, and HIS_352 (Fig. 7B and Supplementary Table S10).

#### Rg and HB assessment

The Rg analysis defined the atomic positioning within the PV-GNProtecta-TLR4 complex along its axis, and was employed to evaluate protein mobility and rigidity through variations in structural compactness [129]. The mean Rg of the PV-GNProtecta-TLR4 assembly was 40.26 Å. Moreover, minor fluctuations during the intermediate phase, particularly around 20 to 25 ns and 70 to 80 ns were observed, which may reflect transient conformational rearrangements (Fig. 7C and Supplementary Table S11).

HB analysis was performed to assess the overall hydrogen-bonding dynamics of the PV-GNProtecta-TLR4 assembly throughout the 100 ns molecular dynamics simulation. The complex exhibited an average hydrogen bond count of 719.50, with values ranging from 670 to 785 during the trajectory (Fig. 7D and Supplementary Table S12). These values represent the global hydrogen-bonding profile of the complete simulated system and do not exclusively correspond to vaccine-TLR4 interface contacts.

#### Post-simulation MM-GBSA

Post-simulation MM-GBSA analysis was performed to compare the binding energetics of the PV-GNProtecta-TLR4 complex before and after the 100 ns molecular dynamics simulation. The calculated binding free energy (ΔG Bind) at the initial (0 ns) and final (100 ns) trajectory frames was − 321.20 kcal/mol and − 201.49 kcal/mol, respectively. These endpoint calculations indicate a difference in the estimated energetic state of the complex after simulation The vaccine-TLR4 complex had an estimated van der Waals (vdW) energy, electrostatic energy, polar solvation energy, non-polar solvation energy of − 270.1168901 kcal/mol, 345.5373371 kcal/mol, − 229.0508384 kcal/mol, − 40.23432613 kcal/mol (Supplementary Fig. S5 and Supplementary Table S13).

#### PCA and DCCM assessment

**Fig. 8:**
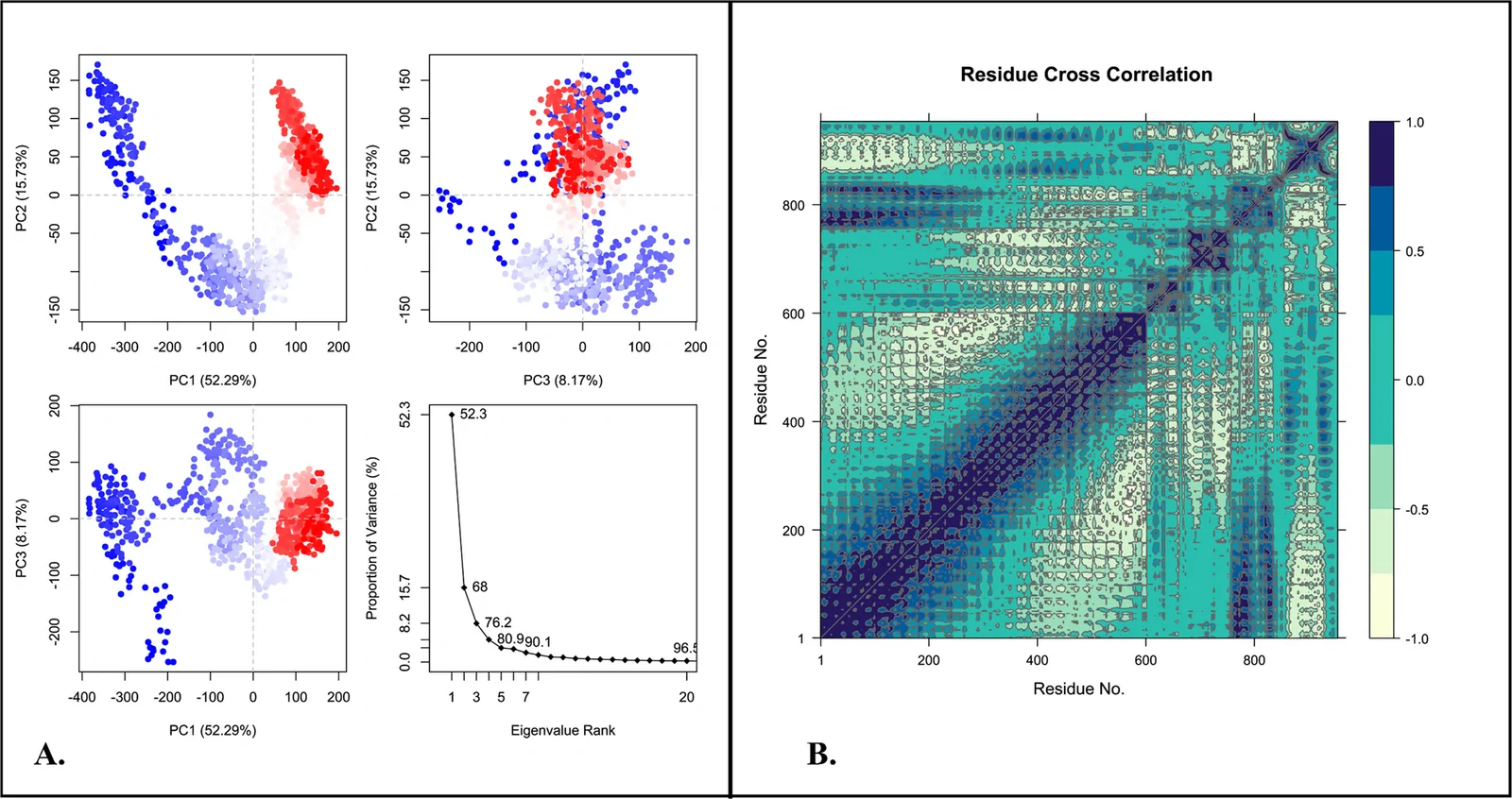
Essential dynamics analysis of the PV-GNProtecta-TLR4 complex obtained from the MD simulation. **A** PCA scatter plots and eigenvalue profile. **B** DCCM heatmap highlighting coordinated and inverse residue fluctuations within the PV-GNProtecta-TLR4 interaction

PCA was carried out to find out the conformational dynamics within the PV-GNProtecta–TLR4 interaction, and the dynamics and structures of the complex were identified by analyzing the first three principal components (PCs). PC1 contributed the highest variance of 52.29%, followed by PC2 with 15.73%, and PC3 with 8.17%, resulting in a cumulative variance of 76.19% (Fig. 8A). The dominant contribution of PC1 indicated that the overall motion of the complex was concentrated within a limited conformational space, reflecting notable structural stability during the simulation. The blue regions represented the highest degree of mobility, whereas the white regions indicated moderate movement, and the red regions corresponded to the lowest level of mobility [130]. DCCM analysis of the PV-GNProtecta–TLR4 complex revealed correlated residue-residue motions throughout the MD simulation [131]. Strong positive correlations were mainly concentrated along the diagonal regions, indicating probable coordinated movements among neighboring residues and stable internal interactions within the complex (Fig. 8B). A few weak anti-correlated regions were also observed, reflecting limited opposing residue fluctuations.

### Optimization of codon and in silico cloning

**Fig. 9:**
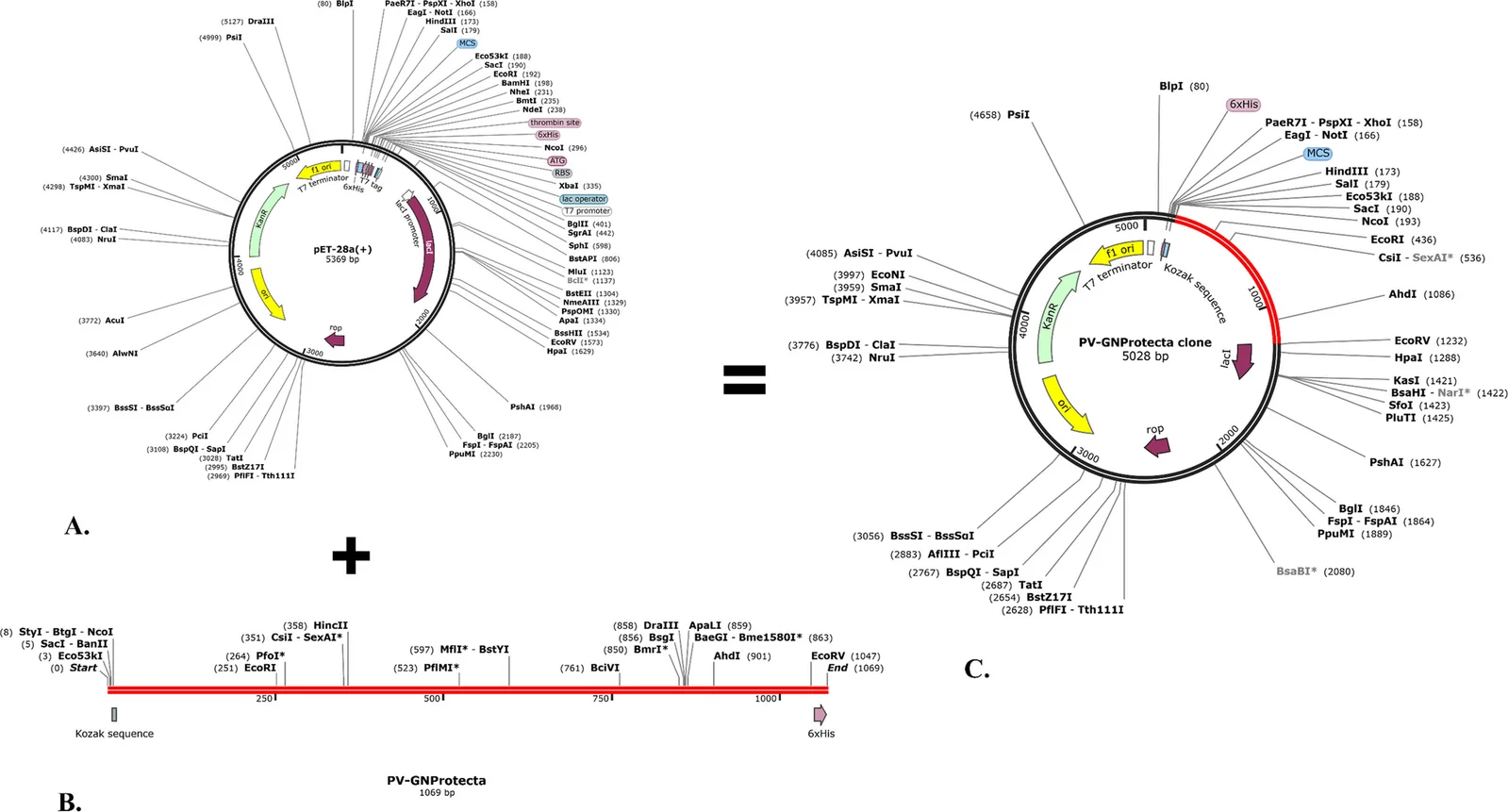
Codon optimization and virtual cloning of the PV-GNProtecta vaccine construct into the pET-28a (+) expression vector. The optimized vaccine region was incorporated into the plasmid backbone through selected restriction enzyme (Eco53KI and EcoRV) sites to generate a recombinant expression construct. **A** pET-28a (+) vector map. **B** Codon-optimized PV-GNProtecta sequence. **C** Recombinant pET-28a (+)-PV-GNProtecta plasmid

The JCat server yielded a codon-adapted DNA sequence for PV-GNProtecta, comprising 1156 nucleotides with a codon adaptation index (CAI) of 1 and a total GC percentage of 46.59%. Subsequently, the recombinant plasmid was constructed through the incorporation of this optimized coding region into the pET-28a (+) vector using SnapGene software (Fig. 9).

### Computational analysis of immune response

**Fig. 10:**
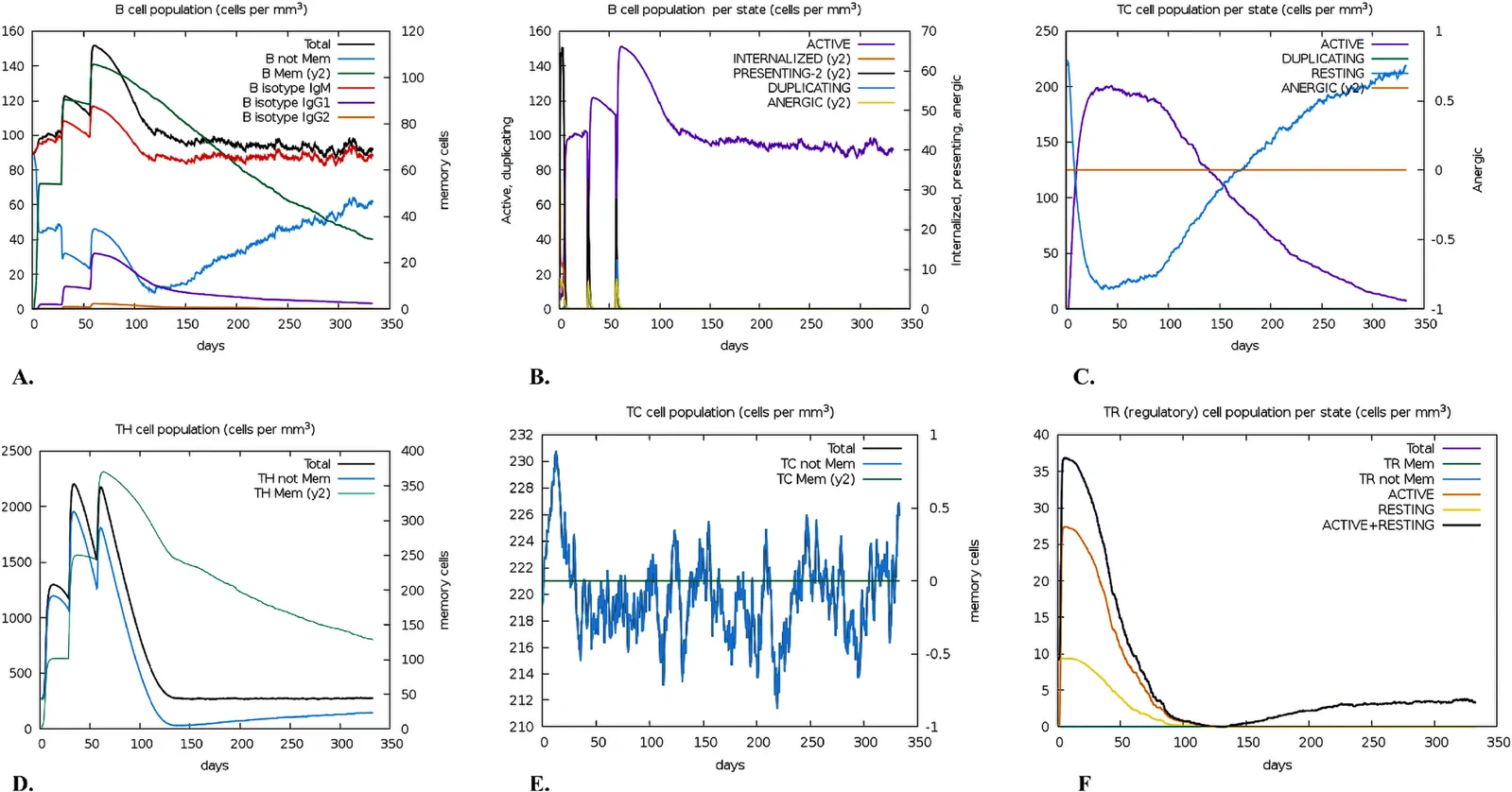
Immune cell population dynamics during the *in-silico* simulation of the PV-GNProtecta vaccine candidate, showing the cellular immune responses throughout the simulation period, including **A** B-cell counts, **B** B-cell counts per state, **C** TC cell counts per state, **D** TH cell counts, **E** TC cell counts, and **F** TR cell counts per state

The in silico immune profiling study further predicted the ability of PV-GNProtecta to promote a potent, coordinated, and persistent immunity throughout the whole observation period. Following the initial and subsequent antigen administrations, antigen levels decreased rapidly, thereby indicating effective immune clearance mediated predominantly through a strong humoral immune response (Supplementary Fig. S6A). IgM levels increased sharply during the early phase of immunization, whereas elevated IgG1 + IgG2 antibody titers were maintained for an extended duration, suggesting the establishment of long-term adaptive immunity (Supplementary Fig. S6A). In parallel, important cytokines, including IL-2 and IFN-γ, exhibited moderate yet distinct transient peaks (Supplementary Fig. S6B). This enhanced humoral activity was accompanied by a progressive transition of B-cell populations from resting states toward active, proliferating, and plasma-cell forms, which continuously supported antibody production over time (Fig. 10A, B). Meanwhile, the cellular component of immunity was predicted to maintain strong and persistent activity throughout the entire simulation, with cytotoxic T-cell populations displaying dynamic changes across active, resting, and anergic states (Fig. 10C). Helper T-cell populations were predicted to maintain sustained activity with considerable memory TH-cell populations throughout the simulation period (Fig. 10D). Additionally, the TC-cell population profile was predicted to support the maintenance of memory TC-cell populations, indicating the potential development of cellular immune memory (Fig. 10E). Regulatory T-cell (TR) populations were predicted to undergo an early transient increase (Fig. 10F), which may contribute to maintaining immune balance during immune activation before gradually returning toward baseline levels. These computational findings suggested that the vaccine construct may provide a rapid, amplified, and protective recall response upon subsequent exposure to the target pathogen.

### Structural modeling of the mRNA vaccine construct

The predicted mRNA construct showed an MFE (Minimum Free Energy) value of − 285.20 kcal/mol within the optimum model and − 239.07 kcal/mol for the centroid structural configuration. Additionally, the thermodynamic ensemble reached − 300.56 kcal/mol. The RNAfold web server provided the MFE structural representation illustrating nucleotide pairing likelihoods (Supplementary Fig. S7A), the MFE folding structure showing positional entropy variations (Supplementary Fig. S7B), centroid structure profile illustrating nucleotide pairing likelihoods (Supplementary Fig. S7C), and centroid structure visualization depicting entropy distribution (Supplementary Fig. S7D).

## Discussion

PUUV is a clinically important zoonotic orthohantavirus responsible for NE, the mildest form of HFRS, predominantly circulating in Northern and Central Europe [9, 17, 132]. Although associated with relatively low mortality compared to other hantaviruses, PUUV infection can lead to acute renal dysfunction, thrombocytopenia, endothelial injury, and immune dysregulation driven by excessive cytokine responses and vascular leakage [9, 132, 133]. Environmental persistence of rodent reservoirs and climate-dependent transmission dynamics further contribute to recurrent outbreaks and sustained public health burden [17, 134]. Despite progress in understanding viral pathogenesis and host interactions, there is still no approved vaccine available, underscoring the importance of developing effective prevention approaches.

Several vaccine development approaches have previously been explored, including nucleoprotein-based multi-epitope vaccines and synthetic M-segment DNA vaccines. Bhattacharya et al. [135] developed a computational multi-epitope vaccine targeting the PUUV nucleoprotein and predicted favorable immunogenicity, population coverage, and receptor-binding characteristics through immunoinformatics analyses [135]. Similarly, Brocato et al. [45] reported a synthetic codon-optimized M-gene DNA vaccine that elicited neutralizing antibodies and conferred protection against PUUV infection in animal models [45]. These approaches were developed using different vaccine platforms and antigen-selection strategies, highlighting the need for further exploration of multi-antigen computational vaccine designs integrating epitope selection, structural assessment, and immunological prediction. Therefore, the development of vaccine candidates with broader antigen representation and enhanced predicted immunological properties remains an important scientific objective.

In this study, PV-GNProtecta was designed and computationally evaluated as an mRNA multi-epitope vaccine candidate incorporating conserved regions from both PUUV G and N proteins. The selection of G and N proteins was based on their complementary biological and immunological relevance. The G protein is involved in viral attachment and entry and represents an important target for antibody recognition, whereas the N protein contributes to viral genome-associated functions and exhibits conservation and immunogenicity among orthohantaviruses [2]. The combined inclusion of these proteins was intended to integrate antibody-accessible viral surface determinants with conserved internal viral components. However, experimental studies are required to determine whether this computational strategy translates into improved immune responses.

Compared with previous PUUV vaccine strategies, Bhattacharya et al. [135] developed a nucleoprotein-based multi-epitope vaccine design using immunoinformatics approaches, whereas PV-GNProtecta incorporates conserved epitopes from both PUUV glycoprotein (G) and nucleoprotein (N) to achieve broader antigen representation. In addition, Brocato et al. [45] evaluated a synthetic M-gene-based DNA vaccine approach, demonstrating the potential of genetic vaccine platforms against PUUV.

In contrast, PV-GNProtecta explores an mRNA multi-epitope framework combined with integrated computational analyses, including epitope prediction, structural modeling, molecular interaction analysis, and immune simulation. Nevertheless, the biological superiority of PV-GNProtecta cannot be established without experimental validation.

Sequence conservation analysis indicated variable conservation patterns among the selected epitopes. While some epitopes displayed relatively higher conservation across the analyzed PUUV sequences, others showed moderate conservation levels. Therefore, epitope selection was based on a combination of conservation analysis and predicted immunological characteristics, including antigenicity, HLA-binding potential, population coverage, and safety-related assessments. The inclusion of MHC I and MHC II, along with B-cell epitopes selected on the basis of predicted antigenicity, non-allergenicity, and non-toxicity, may contribute to the potential induction of coordinated cell-mediated and antibody-mediated immunity [2, 133, 136]. Although additional epitope prediction tools may provide further confirmation, this study used a multi-level validation approach, including sequence, immunological, and structural analyses, to improve the reliability of epitope selection. Compared with the nucleoprotein-only construct reported by Bhattacharya et al. [135], PV-GNProtecta incorporates epitopes from both glycoprotein and nucleoprotein antigens, potentially broadening theoretical immune recognition by targeting both surface entry proteins and conserved internal viral components. These computational predictions should be interpreted as evidence supporting epitope prioritization rather than direct confirmation of immunogenicity, cross-protective immunity, or vaccine efficacy.

Population coverage analysis predicted broad HLA representation across different geographical populations, indicating that the selected epitopes may interact with diverse MHC alleles despite extensive HLA polymorphism, a major challenge in epitope-based vaccine design. Consistent with Bhattacharya et al. [135], high population coverage was observed for the selected epitopes, suggesting theoretical applicability across genetically diverse populations. Furthermore, the incorporation of dual-antigen epitopes from both PUUV G and N proteins may provide broader antigen representation compared with single-antigen vaccine designs. Nevertheless, predicted population coverage reflects computational estimates of HLA distribution and epitope–MHC compatibility; therefore, it should not be interpreted as direct evidence of clinical immunogenicity, vaccine responsiveness, or protective efficacy.

Physicochemical characterization further supported the predicted favorable properties of PV-GNProtecta. The construct suggested favorable physicochemical characteristics, including an instability index of 27.30, aliphatic index of 77.73, GRAVY value of − 0.615, and molecular weight of 38.60 kDa. These parameters suggest potentially favorable solubility, thermostability, and hydrophilic characteristics of the designed construct and indicate its compatibility with computationally predicted recombinant expression approaches. BLASTp analysis against the human proteome revealed no significant sequence similarity between PV-GNProtecta and human proteins. This finding further supports the safety consideration of the vaccine construct by suggesting a reduced possibility of unintended immune cross-reactivity. The predicted high antigenicity together with non-allergenic and non-toxic characteristics supports the prioritization of PV-GNProtecta as a potential vaccine candidate; however, these computational assessments cannot substitute for experimental evaluation of safety and biological performance.

Secondary structure analysis suggested a predominance of α-helices and flexible coil regions, which may enhance epitope accessibility and immune recognition. Collectively, these analyses provide computational support for the physicochemical characterization of PV-GNProtecta, while experimental validation remains necessary to determine its actual biological suitability.

Tertiary structure modeling and refinement predicted a computationally improved structural representation of the PV-GNProtecta construct. The refined model showed improved validation metrics, including 95.5% of residues within favored Ramachandran regions, an improved QMEAN *Z*-score, and a high ERRAT quality score, suggesting enhanced model quality after refinement. These computational validation parameters indicate improved structural plausibility of the predicted model and do not confirm the existence of a biologically stable conformation under physiological conditions. Disulfide engineering analysis identified potential residue positions that may contribute to improved structural rigidity and conformational stability following cysteine-mediated optimization. However, experimental validation and comparative analysis of engineered mutant structures are required to confirm these effects. The effects of these computationally designed modifications require experimental confirmation to determine their actual influence on protein folding, stability, and antigen presentation. Compared with the nucleoprotein-based vaccine design reported by Bhattacharya et al. [135], the present study additionally incorporated computational disulfide optimization as a structural assessment approach; however, the biological relevance of these modifications remains to be experimentally validated.

Conformational B-cell epitope prediction revealed multiple discontinuous epitope regions within the predicted PV-GNProtecta structure, suggesting the presence of potential surface-accessible antigenic regions. Because conformational B-cell epitope prediction is dependent on the accuracy of the computationally generated structural model, these findings should be interpreted as supportive computational evidence rather than direct confirmation of antibody binding, neutralizing activity, or protective immune responses [2, 133]. The inclusion of glycoprotein-derived epitopes may be particularly relevant, as hantavirus neutralizing antibodies predominantly target viral surface glycoproteins, while nucleoprotein-derived epitopes may contribute to broader theoretical antigen representation and cross-strain immune recognition [2, 129]. Experimental studies are required to determine whether these predicted antigenic regions can induce functional antibody responses.

Molecular docking analysis predicted favorable computational interactions between PV-GNProtecta and TLR4, supported by hydrogen bonding, electrostatic interactions, and hydrophobic contacts. TLR4 is an important innate immune receptor involved in pathogen recognition and downstream immune signaling; however, protein–protein docking alone cannot confirm receptor activation, cytokine induction, or adjuvant activity. Therefore, the predicted PV-GNProtecta-TLR4 interaction should be interpreted as a structural hypothesis requiring experimental validation. Similarly, the predicted interactions of selected epitopes with MHC class I and MHC class II molecules provide computational support for epitope prioritization but cannot independently confirm T-cell immunogenicity, which depends on antigen processing, presentation, T-cell receptor recognition, and host-specific immune factors. Consistent with Bhattacharya et al. [135], computational receptor interaction analyses provide preliminary insights into the potential immunological relevance of multi-epitope vaccine constructs.

MM-GBSA analysis estimated favorable interaction energy between the PV-GNProtecta-TLR4 complex, with a calculated binding free energy of − 166.99 kcal/mol under the applied computational conditions. The estimated energy contribution was primarily associated with electrostatic and van der Waals interactions. Additionally, PRODIGY-based binding affinity prediction provided further quantitative support for the PV-GNProtecta-TLR4 interaction. The favorable ΔG and low predicted Kd value indicate favorable binding affinity, complementing the structural and dynamic analyses performed in this study. These values represent model-based computational estimates and should not be interpreted as experimentally measured binding affinities.

Molecular dynamics simulations over 100 ns provided insights into the dynamic behavior and conformational adaptation of the PV-GNProtecta-TLR4 complex under simulated physiological conditions [120]. RMSD, RMSF, radius of gyration, hydrogen bond analysis, PCA, and DCCM analyses provided additional computational insights into conformational changes and interaction patterns throughout the simulation trajectory. Compared with studies relying primarily on docking predictions, the integration of molecular dynamics and MM-GBSA provides a complementary computational assessment of vaccine-receptor interaction stability; however, experimental studies are required to confirm the biological relevance of these predicted interactions.

Immune simulation analysis predicted a multi-phase immune response pattern within the C-ImmSim computational framework. The simulated antigen clearance profile, antibody production patterns, cytokine variations, and immune cell population dynamics provided theoretical insights into the potential immunological behavior of PV-GNProtecta. The predicted transition from early IgM responses to subsequent IgG production suggested possible antibody maturation patterns within the simulation model. Elevated IL-2 and IFN-γ levels indicated a predicted Th1-associated immune profile, which has been implicated in antiviral responses against hantaviruses [9, 133]. Increased simulated populations of B cells, memory-associated cells, cytotoxic T cells, and helper T cells suggested potential immune activation patterns; however, these outputs represent computational predictions and cannot confirm the establishment of durable immune memory, protective recall responses, or clinical efficacy. Consistent with Bhattacharya et al. [135], computational immune simulation supported the potential immunological relevance of the construct; nevertheless, experimental studies are required to determine whether these predicted responses translate into functional immunity. Codon optimization analysis predicted favorable sequence characteristics, with a CAI value of 1.0 and balanced GC content (46.59%), suggesting compatibility with the selected heterologous expression system in *E. coli* [137]. The in silico insertion of the optimized sequence into the pET-28a(+) vector indicated theoretical compatibility with a bacterial recombinant expression strategy. These analyses represent computational assessments of sequence optimization and do not directly demonstrate efficient expression, translational feasibility, or suitability for an mRNA vaccine platform. Additionally, the predicted mRNA structural characteristics provide preliminary insights into sequence behavior but cannot confirm intracellular stability, translation efficiency, or successful vaccine delivery. Consistent with Brocato et al. [45], codon optimization may support efficient heterologous expression [45]; however, further experimental evaluation and platform-specific optimization are required to determine the feasibility of PV-GNProtecta as an mRNA-based vaccine candidate. The present study integrates epitope-based immunoinformatics, structural assessment, immune simulation, and molecular interaction analyses into a unified computational vaccine design framework.

Overall, PV-GNProtecta showed favorable predicted structural characteristics, theoretical immunological potential, and computationally assessed receptor interaction patterns, highlighting its potential as a multi-epitope vaccine candidate against PUUV infection. Compared with previously reported vaccine approaches, including the nucleoprotein-based vaccine design of Bhattacharya et al. [135] and the synthetic DNA vaccine strategy of Brocato et al. [45], PV-GNProtecta incorporates conserved epitopes from both glycoprotein and nucleoprotein antigens while integrating multiple computational analyses, including epitope prioritization, structural refinement, molecular interaction assessment, and immune simulation. These findings suggest that PV-GNProtecta represents a computationally prioritized candidate requiring further experimental investigation rather than an experimentally validated vaccine strategy.

All findings presented in this study are derived from computational analyses and should therefore be interpreted as predictive rather than experimental evidence. In vitro and in vivo studies are required to validate the immunogenicity, safety, receptor interactions, and protective efficacy of PV-GNProtecta before further development toward clinical applications.

## Limitations of the study

Although PV-GNProtecta was designed and evaluated through an integrated computational workflow, several limitations should be considered. All analyses performed in this study are based on computational predictions, and the predicted antigenicity, allergenicity, toxicity, structural properties, and immune responses require experimental validation before biological conclusions can be established. Epitope identification and prioritization depend on the accuracy and assumptions of prediction algorithms, available sequence datasets, selected parameters, and reference databases. Although multiple immunological characteristics were considered during epitope selection, computational predictions cannot fully reproduce antigen processing, presentation, immune recognition, or host-specific immune responses. The 50S ribosomal protein L7/L12 from Mycobacterium TB may be immunostimulatory; however using it as a molecular adjuvant in an mRNA vaccine may have drawbacks. The bacterial protein L7/L12 may cause unexpected or inappropriate immunological responses, increasing reactogenicity. Pre-existing immunity to bacterial antigens may potentially affect the vaccine construct's immunological response. Adding a protein sequence may also impact antigen processing, presentation, and immune responses to epitopes. Thus, producing a bacterial-derived adjuvant in an mRNA vaccine requires rigorous experimental evaluation for safety, off-target immunological effects, and regulatory feasibility. The projected immunostimulatory potential of L7/L12 should not be regarded evidence of increased vaccination effectiveness without in vitro and in vivo validation. The predicted conformational B-cell epitopes are dependent on the accuracy of the computationally generated three-dimensional structure. Therefore, these predictions should be interpreted cautiously until experimentally determined structural information and antibody-binding studies become available. Molecular docking and MM-GBSA analyses provide computational estimates of molecular interactions and energetic properties under specific modeling conditions. These approaches cannot confirm receptor activation, downstream signaling pathways, or experimentally measured binding affinity. Similarly, the biological relevance of the predicted TLR4 interaction requires further experimental investigation. Molecular dynamics simulation results are influenced by simulation parameters, force-field selection, initial structural models, and trajectory length. In this study, a single 100 ns simulation trajectory was analyzed; therefore, additional independent simulations and experimental studies would provide stronger evidence regarding the dynamic behavior and stability of the vaccine–receptor complex. Although endpoint MM-GBSA analysis provided useful insight into energetic changes after MD simulation, the use of limited frames remains a limitation. Future studies using multiple trajectory frames may improve the reliability of binding energy estimation. Moreover, practical considerations associated with mRNA vaccine development, including RNA delivery efficiency, lipid nanoparticle formulation, nucleotide modification, manufacturing feasibility, and storage stability, were not evaluated within this computational framework and require further experimental investigation. The RNAfold-predicted secondary structure and minimum free energy (MFE) alone are insufficient to establish the intracellular stability, translational efficiency, resistance to degradation, or formulation suitability of an mRNA vaccine so, those outcomes were predictive rather than definitive. Finally, immune simulation approaches such as C-ImmSim provide theoretical predictions of immune response patterns but cannot determine actual vaccine-induced immunity, long-term immune memory, safety, or protective efficacy in living organisms. Therefore, in vitro and in vivo validation studies are essential to evaluate antigen expression, immunogenicity, safety, and protective potential of PV-GNProtecta.

## Conclusion

In this study, an integrated immunoinformatics-based approach was employed to design PV-GNProtecta, an mRNA multi-epitope vaccine candidate targeting conserved regions of PUUV G and N proteins. The designed construct incorporated predicted T-cell and B-cell epitopes selected based on antigenicity, allergenicity, toxicity, and conservancy assessments. The major novelty of PV-GNProtecta lies in the incorporation of conserved epitopes from both the PUUV surface glycoprotein and internal nucleoprotein within an mRNA multi-epitope vaccine framework, providing a broader antigen-targeting strategy compared with single-antigen approaches. Structural modeling, molecular docking, molecular dynamics simulation, and immune simulation analyses provided supportive computational insights into the predicted structural characteristics, molecular interactions, and potential immune response patterns of the designed construct. These findings represent computational predictions and should not be interpreted as confirmation of vaccine efficacy, protective immunity, or clinical applicability without experimental validation.

Overall, PV-GNProtecta represents a computationally prioritized vaccine candidate that warrants further investigation through in vitro and in vivo studies to evaluate antigen expression, immunogenicity, safety, and protective potential against PUUV infection.

## Supplementary Information

Supplementary Material 1: Figure S1. Global and regional population-level coverage analysis of the shortlisted T-cell epitopes, incorporated in the PV-GNProtecta. The stacked bar plot illustrates the predicted distribution and cumulative contribution of MHC I, MHC II, and combined MHC I and MHC II epitopes across diverse geographic populations worldwide. Figure S2. Comparative secondary structure composition analysis of the engineered PV-GNProtecta vaccine. Stacked bar charts illustrate the quantitative percentage distribution of alpha helices, extended strands, beta turns, and random coils modeled across GOR4 and SOPMA. Figure S3. Stereochemical and structural quality assessment of the modeled PV-GNProtecta. (A) Ramachandran plot computed via PROCHECK illustrating backbone conformation analysis. (B) ERRAT2 evaluation displaying an overall quality factor of 91.398%. Figure S4. 3D structural modeling and quality verification of the PV-GNProtecta vaccine construct. (A) The initial 3D conformation of the vaccine construct. (B) Quality assessment of the starting structure; the red star indicates the initial QMEAN Z-score of −6.85. (C) The optimized structural conformation after refining. (D) Final validation plot showing that refinement shifted the model into the native-like zone, yielding an improved Z-score of −2.71. Figure S5. Post-simulation MM-GBSA binding free energy characterization of the PV-GNProtecta-TLR4 complex. The radar plot illustrates the comparative contributions of different energy components, including Coulombic, covalent, hydrogen bonding, lipophilic, packing, solvation energies, and van der Waals at 0 and 100 ns of the simulation. Figure S6. In silico immune profiling study results illustrating the humoral immunogenicity provoked by the PV-GNProtecta vaccine construct, including (A) eradication of antigens and antibody generation and (B) cytokine responses, upon multiple antigen exposures. Figure S7. Predicted mRNA secondary structural models of the PV-GNProtecta vaccine construct showing centroid and MFE structures with positional entropy and base-pair probability distributions. (A and C) Structures colored according to nucleotide pairing likelihoods. (B and D) Illustrate positional entropy within the mRNA structures.

Supplementary Material 2: Table S1. Characterization of PUUV G and N proteins before epitope prediction. Table S2. List of prioritized MHC I, MHC II, and linear B-cell epitopes with their corresponding HLA allele(s) prediction. Table S3. Comprehensive sequence conservation analysis of selected MHC I, MHC II, and linear B-cell epitopes. Table S4. Comparative analysis of secondary structure prediction of PV-GNProtecta using GOR4, SOPMA, and PSIPRED. Table S5. Predicted disulfide bonds in the designed vaccine structure by the Disulfide by Design 2 v2.13 website. Table S6. Predicted conformational B-cell epitopes with the residues and scores. Table S7. Cluster-wise distribution of docking conformations and corresponding energy scores identified during molecular docking analysis. Table S8. PRODIGY-based binding affinity prediction of the PV-GNProtecta–TLR4 complex. Table S9. Protein RMSD. Table S10. Protein RMSF. Table S11. Radius of Gyration. Table S12. Number of Hydrogen Bonds. Table S13. Post-simulation MM-GBSA. Table S14. Comparative assessment of PUUV proteins supporting the selection of G and N proteins for vaccine design.

## Data Availability

Data is contained within the article and Supplementary Material.
