# Supplementary material for: Design and in silico evaluation of PV-GNProtecta: an mRNA multi-epitope vaccine candidate against Puumala virus using immunoinformatics and structural analysis
Source: Genomics Inform. 2026 Oct 12;24:19. doi: 10.1186/s44342-026-00081-8 (PMC13656458; doi:10.1186/s44342-026-00081-8)
Supplement: Supplementary file 2 [file 44342_2026_81_MOESM2_ESM.zip › Supplementary Table 8.docx]

**Supplementary Table 8.** PRODIGY-based binding affinity prediction of the PV-GNProtecta-TLR4 complex

| **Complex** | **ΔG (kcal/mol)** | **Predicted Kd (M)** | **Temperature** |
| --- | --- | --- | --- |
| PV-GNProtecta-TLR4 | -17.6 | 1.3 × 10^-^¹³ | 25°C |
