# Supplementary material for: Design and in silico evaluation of PV-GNProtecta: an mRNA multi-epitope vaccine candidate against Puumala virus using immunoinformatics and structural analysis
Source: Genomics Inform. 2026 Oct 12;24:19. doi: 10.1186/s44342-026-00081-8 (PMC13656458; doi:10.1186/s44342-026-00081-8)
Supplement: Supplementary file 2 [file 44342_2026_81_MOESM2_ESM.zip › Supplementary Table 7.docx]

**Supplementary Table 7.** Cluster-wise distribution of docking conformations and corresponding energy scores identified during molecular docking analysis

| **Cluster** | **Members** | **Representative** | **Weighted Score** |
| --- | --- | --- | --- |
| 0 | 43 | Center | -1023.2 |
|  |  | Lowest Energy | -1023.2 |
| 1 | 36 | Center | -892.6 |
|  |  | Lowest Energy | -918.4 |
| 2 | 30 | Center | -844.3 |
|  |  | Lowest Energy | -983.9 |
| 3 | 27 | Center | -825.2 |
|  |  | Lowest Energy | -851.3 |
| 4 | 27 | Center | -782 |
|  |  | Lowest Energy | -889.3 |
| 5 | 27 | Center | -944.8 |
|  |  | Lowest Energy | -944.8 |
| 6 | 24 | Center | -859.2 |
|  |  | Lowest Energy | -1006.6 |
| 7 | 23 | Center | -947.2 |
|  |  | Lowest Energy | -947.2 |
| 8 | 20 | Center | -938.3 |
|  |  | Lowest Energy | -938.3 |
| 9 | 18 | Center | -829.7 |
|  |  | Lowest Energy | -888.8 |
| 10 | 17 | Center | -1060.6 |
|  |  | Lowest Energy | -1060.6 |
| 11 | 16 | Center | -955.5 |
|  |  | Lowest Energy | -955.5 |
| 12 | 16 | Center | -828.9 |
|  |  | Lowest Energy | -896.6 |
| 13 | 16 | Center | -932.8 |
|  |  | Lowest Energy | -932.8 |
| 14 | 14 | Center | -826.2 |
|  |  | Lowest Energy | -936 |
| 15 | 14 | Center | -925.8 |
|  |  | Lowest Energy | -925.8 |
| 16 | 14 | Center | -925.1 |
|  |  | Lowest Energy | -994.5 |
| 17 | 14 | Center | -887.2 |
|  |  | Lowest Energy | -921.8 |
| 18 | 14 | Center | -825.8 |
|  |  | Lowest Energy | -827.4 |
| 19 | 13 | Center | -834 |
|  |  | Lowest Energy | -940.4 |
| 20 | 12 | Center | -903.3 |
|  |  | Lowest Energy | -1126 |
| 21 | 12 | Center | -871.1 |
|  |  | Lowest Energy | -883.2 |
| 22 | 11 | Center | -819.6 |
|  |  | Lowest Energy | -918.6 |
| 23 | 11 | Center | -790.8 |
|  |  | Lowest Energy | -884.9 |
| 24 | 10 | Center | -946.6 |
|  |  | Lowest Energy | -946.6 |
| 25 | 10 | Center | -944.1 |
|  |  | Lowest Energy | -944.1 |
| 26 | 10 | Center | -858.8 |
|  |  | Lowest Energy | -858.8 |
| 27 | 10 | Center | -852.9 |
|  |  | Lowest Energy | -852.9 |
| 28 | 10 | Center | -851.4 |
|  |  | Lowest Energy | -851.4 |
| 29 | 10 | Center | -832.1 |
|  |  | Lowest Energy | -832.1 |
