# Supplementary material for: Design and in silico evaluation of PV-GNProtecta: an mRNA multi-epitope vaccine candidate against Puumala virus using immunoinformatics and structural analysis
Source: Genomics Inform. 2026 Oct 12;24:19. doi: 10.1186/s44342-026-00081-8 (PMC13656458; doi:10.1186/s44342-026-00081-8)
Supplement: Supplementary file 2 [file 44342_2026_81_MOESM2_ESM.zip › Supplementary Table 6.docx]

**Supplementary Table 6.** Predicted conformational B-cell epitopes with the residues and scores.

| **No.** | **Residues** | **Number of residues** | **Score** |
| --- | --- | --- | --- |
| 1 | A:H347, A:H348, A:H349, A:H350, A:H351, A:H352 | 6 | 0.967 |
| 2 | A:K164, A:A166, A:E167, A:I168, A:Q169, A:H170, A:L171, A:G172, A:H173, A:W174 | 10 | 0.878 |
| 3 | A:A1, A:K2, A:F3, A:V4, A:A5, A:A6, A:W7, A:T8, A:L9, A:K10, A:A11, A:A12, A:A13, A:E14, A:A15, A:A16, A:A17, A:K18, A:M19, A:A20, A:K21, A:L22, A:S23, A:T24, A:D25, A:E26, A:L27, A:L28, A:D29, A:A30, A:F31, A:K32, A:E33, A:M34, A:T35, A:L36, A:L37, A:E38, A:L39 | 39 | 0.856 |
| 4 | A:D243, A:I244, A:N245, A:G246, A:I247, A:R248, A:R249 | 7 | 0.785 |
| 5 | A:E324, A:S327, A:K328, A:K330, A:Q331, A:A332, A:L333, A:I334, A:D335, A:Q336, A:K337, A:V338, A:K339, A:E340, A:I341, A:S342, A:N343, A:Q344 | 18 | 0.719 |
| 6 | A:A61, A:A62, A:A63, A:G64, A:A65, A:A66, A:P67, A:A68, A:G69, A:A70, A:A71, A:V72 | 12 | 0.675 |
| 7 | A:K311, A:D312, A:P313, A:T314, A:G315, A:I316, A:E317, A:P318, A:D319, A:D320, A:K323 | 11 | 0.651 |
| 8 | A:M297, A:S298, A:T299, A:P300, A:T301, A:G302, A:M303, A:K304, A:C305, A:P306, A:E307, A:L308, A:K309 | 13 | 0.646 |
| 9 | A:K218, A:C219, A:C220, A:S221, A:G222, A:I223, A:I224, A:P225, A:M226, A:K227, A:T228, A:D229, A:L230, A:E231 | 14 | 0.642 |
| 10 | A:K255, A:D256, A:P257, A:T258, A:G259, A:I260, A:E261, A:P262, A:D263, A:D264, A:K267, A:E268, A:S270, A:K271 | 14 | 0.641 |
| 11 | A:R178, A:P179, A:K180, A:H181, A:Y183 | 5 | 0.638 |
| 12 | A:A88, A:C89, A:D90, A:K91, A:K92, A:I93, A:G94, A:K97, A:R100, A:A140, A:A141, A:C142, A:A143 | 13 | 0.587 |
| 13 | A:K292, A:P293, A:G294, A:D295, A:I296 | 5 | 0.526 |
