# Supplementary material for: Design and in silico evaluation of PV-GNProtecta: an mRNA multi-epitope vaccine candidate against Puumala virus using immunoinformatics and structural analysis
Source: Genomics Inform. 2026 Oct 12;24:19. doi: 10.1186/s44342-026-00081-8 (PMC13656458; doi:10.1186/s44342-026-00081-8)
Supplement: Supplementary file 2 [file 44342_2026_81_MOESM2_ESM.zip › Supplementary Table 5.docx]

**Supplementary Table 5.** Predicted disulfide bonds in the designed vaccine structure by the Disulfide by Design 2 v2.13 website.

| **Res1 Chain** | **Res1 Seq #** | **Res1 AA** | **Res2 Chain** | **Res2 Seq #** | **Res2 AA** | **Chi3** | **Energy** | **Sum B-Factors** |
| --- | --- | --- | --- | --- | --- | --- | --- | --- |
| A | 67 | PRO | A | 68 | ALA | 118.25 | 2.03 | 60.52 |
| A | 80 | GLU | A | 150 | ALA | -85.82 | 4.34 | 163.87 |
| A | 81 | PHE | A | 131 | ALA | 99.74 | 0.94 | 187.68 |
| A | 83 | VAL | A | 123 | LEU | 115.5 | 6.66 | 187.62 |
| A | 89 | GLY | A | 142 | GLY | 75.01 | 4.19 | 187.09 |
| A | 91 | LYS | A | 142 | GLY | 118.66 | 5.57 | 188.12 |
| A | 171 | LEU | A | 172 | GLY | 126.57 | 5.91 | 67.85 |
| A | 189 | LYS | A | 190 | GLY | 121.08 | 5.68 | 68.74 |
| A | 219 | LYS | A | 220 | GLY | 100.12 | 2.71 | 64.45 |
