# Supplementary material for: Design and in silico evaluation of PV-GNProtecta: an mRNA multi-epitope vaccine candidate against Puumala virus using immunoinformatics and structural analysis
Source: Genomics Inform. 2026 Oct 12;24:19. doi: 10.1186/s44342-026-00081-8 (PMC13656458; doi:10.1186/s44342-026-00081-8)
Supplement: Supplementary file 2 [file 44342_2026_81_MOESM2_ESM.zip › Supplementary Table 3.docx]

**Supplementary Table 3.** Comprehensive sequence conservation analysis of selected MHC I, MHC II, and linear B-cell epitopes.

| **Protein** | **Epitope** | **Percent of protein sequence similarity at identity <= 100%** | **Lower identity cutoff** | **Upper identity cutoff** |
| --- | --- | --- | --- | --- |
| G | LPSSASYTY | 96.77% (90/93) | 88.89% | 100.00% |
|  | AEIQHLGHW | 100.00% (93/93) | 100.00% | 100.00% |
|  | HGSGIIPMKTDLELD | 52.69% (49/93) | 80.00% | 100.00% |
|  | GSGIIPMKTDLELDF | 52.69% (49/93) | 80.00% | 100.00% |
|  | PGEIEKTTQCTVFCTL | 87.10% (81/93) | 87.50% | 100.00% |
|  | PGDIMSTPTGMKCPEL | 38.71% (36/93) | 81.25% | 100.00% |
| N | RPKHLYVSM | 99.00% (99/100) | 88.89% | 100.00% |
|  | GLFPTQIQV | 98.00% (98/100) | 88.89% | 100.00% |
|  | DTSFEDINGIRRPKH | 97.00% (97/100) | 86.67% | 100.00% |
|  | DPTGIEPDDHLKERS | 100.00% (100/100) | 100.00% | 100.00% |
|  | DPTGIEPDDHLKERSS | 100.00% (100/100) | 100.00% | 100.00% |
|  | QALIDQKVKEISNQEP | 51.00% (51/100) | 93.75% | 100.00% |
