# Supplementary material for: Design and in silico evaluation of PV-GNProtecta: an mRNA multi-epitope vaccine candidate against Puumala virus using immunoinformatics and structural analysis
Source: Genomics Inform. 2026 Oct 12;24:19. doi: 10.1186/s44342-026-00081-8 (PMC13656458; doi:10.1186/s44342-026-00081-8)
Supplement: Supplementary file 2 [file 44342_2026_81_MOESM2_ESM.zip › Supplementary Table 4.docx]

**Supplementary Table 4.** Comparative analysis of secondary structure prediction of PV-GNProtecta using GOR4, SOPMA, and PSIPRED

| **Prediction Server** | **α-Helix (%)** | **β-Strand (%)** | **Random Coil (%)** | **Summary of Prediction** |
| --- | --- | --- | --- | --- |
| GOR4 | 53.41 | 11.93 | 34.66 | Predicted α-helices as the predominant secondary structure element, followed by coil regions and β-strands |
| SOPMA | 44.32 | 8.24 | 42.90 | Predicted a similar distribution pattern with α-helices and coil regions as the major components |
| PSIPRED | 43.47 | 11.65 | 44.89 | Predicted a comparable secondary structure composition consisting of helices, strands, and coil regions |
| **Overall observation** | -- | -- | -- | The three prediction methods showed a comparable overall secondary structure pattern, with differences in the predicted proportions among individual structural elements |
