# Supplementary material for: Design and in silico evaluation of PV-GNProtecta: an mRNA multi-epitope vaccine candidate against Puumala virus using immunoinformatics and structural analysis
Source: Genomics Inform. 2026 Oct 12;24:19. doi: 10.1186/s44342-026-00081-8 (PMC13656458; doi:10.1186/s44342-026-00081-8)
Supplement: Supplementary file 2 [file 44342_2026_81_MOESM2_ESM.zip › Supplementary Table 2.docx]

**Supplementary Table 2.** List of prioritized MHC I, MHC II, and linear B-cell epitopes with their corresponding HLA allele(s) prediction.

| **Protein** | **Epitope** | **Peptide** | **Corresponding HLA allele(s)** |
| --- | --- | --- | --- |
| **G** | MHC I | LPSSASYTY | HLA-B*35:01; HLA-B*53:01; HLA-A*01:01; HLA-A*26:01; HLA-B*51:01; HLA-A*30:02; HLA-B*07:02; HLA-B*58:01; HLA-B*15:01; HLA-B*44:03; HLA-B*44:02; HLA-B*57:01; HLA-A*32:01; HLA-A*68:01; HLA-B*08:01; HLA-A*24:02; HLA-A*11:01; HLA-A*23:01; HLA-A*03:01; HLA-A*33:01 |
|  |  | AEIQHLGHW | HLA-B*44:03; HLA-B*44:02; HLA-B*40:01; HLA-B*58:01; HLA-B*53:01; HLA-B*57:01; HLA-A*26:01; HLA-A*32:01; HLA-B*15:01; HLA-A*23:01; HLA-A*30:02; HLA-A*24:02; HLA-B*35:01; HLA-A*01:01; HLA-B*07:02; HLA-A*02:06; HLA-B*51:01; HLA-A*11:01; HLA-B*08:01; HLA-A*02:03 |
|  | MHC II | HGSGIIPMKTDLELD | HLA-DRB4*01:01; HLA-DRB1*04:05; HLA-DQA1*03:01/DQB1*03:02; HLA-DRB1*04:01; HLA-DRB1*12:01; HLA-DQA1*01:01/DQB1*05:01; HLA-DQA1*05:01/DQB1*02:01; HLA-DRB1*08:02; HLA-DRB1*15:01; HLA-DRB1*11:01; HLA-DRB3*02:02; HLA-DRB1*03:01 |
|  |  | GSGIIPMKTDLELDF | HLA-DRB4*01:01; HLA-DRB1*04:05; HLA-DQA1*03:01/DQB1*03:02; HLA-DRB1*04:01; HLA-DRB1*12:01; HLA-DQA1*01:01/DQB1*05:01; HLA-DQA1*05:01/DQB1*02:01; HLA-DRB1*08:02; HLA-DRB1*15:01; HLA-DRB1*11:01; HLA-DRB3*01:01; HLA-DRB1*03:01 |
|  | B-cell | PGEIEKTTQCTVFCTL | N/A (B-cell epitope; HLA presentation not applicable) |
|  |  | PGDIMSTPTGMKCPEL | N/A (B-cell epitope; HLA presentation not applicable) |
| **N** | MHC I | RPKHLYVSM | HLA-B*07:02; HLA-B*08:01; HLA-B*35:01; HLA-B*51:01; HLA-B*53:01; HLA-A*30:01; HLA-B*15:01; HLA-A*26:01; HLA-B*57:01; HLA-A*30:02; HLA-A*32:01; HLA-A*68:02; HLA-B*58:01; HLA-A*31:01; HLA-A*33:01; HLA-A*23:01; HLA-A*01:01; HLA-B*40:01; HLA-A*24:02; HLA-B*44:02 |
|  |  | GLFPTQIQV | HLA-A*02:01; HLA-A*02:03; HLA-A*02:06; HLA-A*32:01; HLA-A*68:02; HLA-A*30:01; HLA-B*15:01; HLA-A*30:02; HLA-A*03:01; HLA-B*51:01; HLA-A*31:01; HLA-B*57:01; HLA-B*08:01; HLA-B*58:01; HLA-B*40:01; HLA-A*26:01; HLA-A*11:01; HLA-A*01:01; HLA-A*23:01; HLA-A*33:01 |
|  | MHC II | DTSFEDINGIRRPKH | HLA-DRB5*01:01; HLA-DPA1*02:01/DPB1*05:01; HLA-DRB1*13:02; HLA-DRB1*01:01; HLA-DPA1*02:01/DPB1*01:01; HLA-DRB3*01:01; HLA-DRB3*02:02; HLA-DRB1*11:01; HLA-DRB1*07:01; HLA-DRB1*04:01 |
|  |  | DPTGIEPDDHLKERS | HLA-DRB1*03:01; HLA-DRB3*01:01; HLA-DRB1*13:02; HLA-DQA1*01:01/DQB1*05:01; HLA-DRB4*01:01; HLA-DPA1*02:01/DPB1*01:01; HLA-DQA1*05:01/DQB1*02:01; HLA-DPA1*01:03/DPB1*04:01; HLA-DRB3*02:02; HLA-DRB1*04:01 |
|  | B-cell | DPTGIEPDDHLKERSS | N/A (B-cell epitope; HLA presentation not applicable) |
|  |  | QALIDQKVKEISNQEP | N/A (B-cell epitope; HLA presentation not applicable) |
