# Supplementary material for: Design and in silico evaluation of PV-GNProtecta: an mRNA multi-epitope vaccine candidate against Puumala virus using immunoinformatics and structural analysis
Source: Genomics Inform. 2026 Oct 12;24:19. doi: 10.1186/s44342-026-00081-8 (PMC13656458; doi:10.1186/s44342-026-00081-8)
Supplement: Supplementary file 2 [file 44342_2026_81_MOESM2_ESM.zip › Supplementary Table 14.docx]

**Supplementary Table 14.** Comparative assessment of PUUV proteins supporting the selection of G and N proteins for vaccine design

| **Criteria** | **Glycoprotein (G)** | **Nucleoprotein (N)** | **RNA-dependent RNA polymerase (RdRp/L)** |
| --- | --- | --- | --- |
| Major biological function | Mediates viral attachment, entry, and membrane fusion | Involved in viral genome packaging, replication, and assembly | Responsible for viral RNA replication and transcription |
| Immunological importance | Surface protein and major target of neutralizing antibodies | Highly immunogenic protein inducing strong immune responses during infection | Mainly involved in intracellular replication processes |
| Accessibility to immune recognition | Highly accessible due to surface localization | Recognized by cellular and humoral immune responses | Limited exposure to antibody-mediated immunity |
| Vaccine relevance | Provides potential B-cell and T-cell epitopes | Provides conserved B-cell and T-cell epitopes | Less suitable for antibody-focused vaccine strategies |
| Sequence conservation | Conserved regions identified among PUUV variants | Highly conserved among PUUV strains | Conserved enzyme region but less prioritized for immune targeting |
| Epitope-based evaluation | Selected epitopes showed antigenic, non-allergenic, and non-toxic properties | Selected epitopes showed antigenic, non-allergenic, and non-toxic properties | Not included in epitope screening due to lower suitability as vaccine target |
| Overall suitability | High | High | Moderate |
