# Supplementary material for: Design and in silico evaluation of PV-GNProtecta: an mRNA multi-epitope vaccine candidate against Puumala virus using immunoinformatics and structural analysis
Source: Genomics Inform. 2026 Oct 12;24:19. doi: 10.1186/s44342-026-00081-8 (PMC13656458; doi:10.1186/s44342-026-00081-8)
Supplement: Supplementary file 2 [file 44342_2026_81_MOESM2_ESM.zip › Supplementary Table 13.docx]

**Supplementary Table 13.** Post Simulation MM-GBSA.

| **Title** | **PV-GNProtecta-TLR4** | |
| --- | --- | --- |
|  | **0 ns** | **100 ns** |
| **r_psp_MMGBSA_dG_Bind** | -321.1976102 | -201.4952234 |
| **r_psp_MMGBSA_dG_Bind_Coulomb** | -159.9760089 | 345.5373371 |
| **r_psp_MMGBSA_dG_Bind_Covalent** | 65.2233478 | 10.34479925 |
| **r_psp_MMGBSA_dG_Bind_Hbond** | -27.70638387 | -14.19739494 |
| **r_psp_MMGBSA_dG_Bind_Lipo** | -70.9692479 | -40.23432613 |
| **r_psp_MMGBSA_dG_Bind_Packing** | -5.057422033 | -3.799805184 |
| **r_psp_MMGBSA_dG_Bind_Solv_GB** | 216.4141901 | -229.0508384 |
| **r_psp_MMGBSA_dG_Bind_vdW** | -339.1247693 | -270.1168901 |
