# Supplementary material for: Design and in silico evaluation of PV-GNProtecta: an mRNA multi-epitope vaccine candidate against Puumala virus using immunoinformatics and structural analysis
Source: Genomics Inform. 2026 Oct 12;24:19. doi: 10.1186/s44342-026-00081-8 (PMC13656458; doi:10.1186/s44342-026-00081-8)
Supplement: Supplementary file 2 [file 44342_2026_81_MOESM2_ESM.zip › Supplementary Table 1.docx]

**Supplementary Table 1.** Characterization of PUUV G and N proteins before epitope prediction.

| **Protein** | **Signal peptide (SignalP 6.0)** | **Transmembrane regions (DeepTMHMM)** | **Subcellular localization (UniProt)** |
| --- | --- | --- | --- |
| G | Present; cleavage site between residues 24-25 | Three predicted regions (495-515, 637-653, 1115-1132) | Virion membrane, host cell surface, ER and Golgi membrane |
| N | Not detected | One predicted region (120-129) | Virion, host cytoplasm, perinuclear region, Golgi-associated |
