# Supplementary material for: Design and in silico evaluation of PV-GNProtecta: an mRNA multi-epitope vaccine candidate against Puumala virus using immunoinformatics and structural analysis
Source: Genomics Inform. 2026 Oct 12;24:19. doi: 10.1186/s44342-026-00081-8 (PMC13656458; doi:10.1186/s44342-026-00081-8)
Supplement: Supplementary file 2 [file 44342_2026_81_MOESM2_ESM.zip › Supplementary Table 11.docx]

**Supplementary Table 11.** Radius of Gyration.

| **TimeStep** | **PV-GNProtecta-TLR4** |
| --- | --- |
| 0 | 41.574 |
| 100.002 | 41.466 |
| 200.004 | 41.585 |
| 300.006 | 41.767 |
| 400.008 | 41.394 |
| 500.01 | 41.325 |
| 600.012 | 41.155 |
| 700.014 | 41.166 |
| 800.016 | 41.628 |
| 900.018 | 41.819 |
| 1000.02 | 42.33 |
| 1100.022 | 42.77 |
| 1200.024 | 42.371 |
| 1300.026 | 42.968 |
| 1400.028 | 43.006 |
| 1500.03 | 42.955 |
| 1600.032 | 42.685 |
| 1700.034 | 42.317 |
| 1800.036 | 42.053 |
| 1900.038 | 41.959 |
| 2000.04 | 42.509 |
| 2100.042 | 41.941 |
| 2200.044 | 41.706 |
| 2300.046 | 41.938 |
| 2400.048 | 41.835 |
| 2500.05 | 41.818 |
| 2600.052 | 41.939 |
| 2700.054 | 41.712 |
| 2800.056 | 41.957 |
| 2900.058 | 41.756 |
| 3000.06 | 41.827 |
| 3100.062 | 42.195 |
| 3200.064 | 41.903 |
| 3300.066 | 41.688 |
| 3400.068 | 41.444 |
| 3500.07 | 41.491 |
| 3600.072 | 41.354 |
| 3700.074 | 41.516 |
| 3800.076 | 41.474 |
| 3900.078 | 41.779 |
| 4000.08 | 42.217 |
| 4100.082 | 42.033 |
| 4200.084 | 41.892 |
| 4300.086 | 41.747 |
| 4400.088 | 41.653 |
| 4500.09 | 41.152 |
| 4600.092 | 41.419 |
| 4700.094 | 41.081 |
| 4800.096 | 41.194 |
| 4900.098 | 40.66 |
| 5000.1 | 39.988 |
| 5100.102 | 40.281 |
| 5200.104 | 40.427 |
| 5300.106 | 40.505 |
| 5400.108 | 40.655 |
| 5500.11 | 40.323 |
| 5600.112 | 40.592 |
| 5700.114 | 40.354 |
| 5800.116 | 40.036 |
| 5900.118 | 40.149 |
| 6000.12 | 40.383 |
| 6100.122 | 40.01 |
| 6200.124 | 40.191 |
| 6300.126 | 43.987 |
| 6400.128 | 40.87 |
| 6500.13 | 40.753 |
| 6600.132 | 40.93 |
| 6700.134 | 41.212 |
| 6800.136 | 40.895 |
| 6900.138 | 41.504 |
| 7000.14 | 40.86 |
| 7100.142 | 41.183 |
| 7200.144 | 41.139 |
| 7300.146 | 41.588 |
| 7400.148 | 41.024 |
| 7500.15 | 40.883 |
| 7600.152 | 40.979 |
| 7700.154 | 40.782 |
| 7800.156 | 40.474 |
| 7900.158 | 40.347 |
| 8000.16 | 40.547 |
| 8100.162 | 40.617 |
| 8200.164 | 40.08 |
| 8300.166 | 40.071 |
| 8400.168 | 39.871 |
| 8500.17 | 40.112 |
| 8600.172 | 40.67 |
| 8700.174 | 40.069 |
| 8800.176 | 39.863 |
| 8900.178 | 39.979 |
| 9000.18 | 40.167 |
| 9100.182 | 39.618 |
| 9200.184 | 39.447 |
| 9300.186 | 39.285 |
| 9400.188 | 39.388 |
| 9500.19 | 39.75 |
| 9600.192 | 39.848 |
| 9700.194 | 39.347 |
| 9800.196 | 39.172 |
| 9900.198 | 39.503 |
| 10000.2 | 39.342 |
| 10100.202 | 39.353 |
| 10200.204 | 39.494 |
| 10300.206 | 39.21 |
| 10400.208 | 38.956 |
| 10500.21 | 38.905 |
| 10600.212 | 38.923 |
| 10700.214 | 39.02 |
| 10800.216 | 39.401 |
| 10900.218 | 39.606 |
| 11000.22 | 39.704 |
| 11100.222 | 40.083 |
| 11200.224 | 40.664 |
| 11300.226 | 40.58 |
| 11400.228 | 40.248 |
| 11500.23 | 40.49 |
| 11600.232 | 40.727 |
| 11700.234 | 40.322 |
| 11800.236 | 40.276 |
| 11900.238 | 40.187 |
| 12000.24 | 39.893 |
| 12100.242 | 40.376 |
| 12200.244 | 40.157 |
| 12300.246 | 39.798 |
| 12400.248 | 39.757 |
| 12500.25 | 39.717 |
| 12600.252 | 39.643 |
| 12700.254 | 39.377 |
| 12800.256 | 39.639 |
| 12900.258 | 39.727 |
| 13000.26 | 39.561 |
| 13100.262 | 39.75 |
| 13200.264 | 39.924 |
| 13300.266 | 40.048 |
| 13400.268 | 40.016 |
| 13500.27 | 39.86 |
| 13600.272 | 39.995 |
| 13700.274 | 40.519 |
| 13800.276 | 40.576 |
| 13900.278 | 39.874 |
| 14000.28 | 40.029 |
| 14100.282 | 39.844 |
| 14200.284 | 39.835 |
| 14300.286 | 40.116 |
| 14400.288 | 40.133 |
| 14500.29 | 39.969 |
| 14600.292 | 39.958 |
| 14700.294 | 40.487 |
| 14800.296 | 41.013 |
| 14900.298 | 40.639 |
| 15000.3 | 40.379 |
| 15100.302 | 40.783 |
| 15200.304 | 40.861 |
| 15300.306 | 41.204 |
| 15400.308 | 41.131 |
| 15500.31 | 40.941 |
| 15600.312 | 41.459 |
| 15700.314 | 41.104 |
| 15800.316 | 41.378 |
| 15900.318 | 41.509 |
| 16000.32 | 41.577 |
| 16100.322 | 41.638 |
| 16200.324 | 41.101 |
| 16300.326 | 40.506 |
| 16400.328 | 40.262 |
| 16500.33 | 40.32 |
| 16600.332 | 40.176 |
| 16700.334 | 39.873 |
| 16800.336 | 40.115 |
| 16900.338 | 40.294 |
| 17000.34 | 40.885 |
| 17100.342 | 40.713 |
| 17200.344 | 40.678 |
| 17300.346 | 40.476 |
| 17400.348 | 40.574 |
| 17500.35 | 40.393 |
| 17600.352 | 40.426 |
| 17700.354 | 40.363 |
| 17800.356 | 40.498 |
| 17900.358 | 40.793 |
| 18000.36 | 40.864 |
| 18100.362 | 40.282 |
| 18200.364 | 40.473 |
| 18300.366 | 40.132 |
| 18400.368 | 40.11 |
| 18500.37 | 40.195 |
| 18600.372 | 40.735 |
| 18700.374 | 40.751 |
| 18800.376 | 40.808 |
| 18900.378 | 40.764 |
| 19000.38 | 44.71 |
| 19100.382 | 41.441 |
| 19200.384 | 45.077 |
| 19300.386 | 41.129 |
| 19400.388 | 41.987 |
| 19500.39 | 41.562 |
| 19600.392 | 41.383 |
| 19700.394 | 41.495 |
| 19800.396 | 41.481 |
| 19900.398 | 45.585 |
| 20000.4 | 45.02 |
| 20100.402 | 45.36 |
| 20200.404 | 41.593 |
| 20300.406 | 45.151 |
| 20400.408 | 41.009 |
| 20500.41 | 44.996 |
| 20600.412 | 44.593 |
| 20700.414 | 44.733 |
| 20800.416 | 45.093 |
| 20900.418 | 41.451 |
| 21000.42 | 45.17 |
| 21100.422 | 41.065 |
| 21200.424 | 41.245 |
| 21300.426 | 41.551 |
| 21400.428 | 41.553 |
| 21500.43 | 41.62 |
| 21600.432 | 41.201 |
| 21700.434 | 41.3 |
| 21800.436 | 41.761 |
| 21900.438 | 45.524 |
| 22000.44 | 45.46 |
| 22100.442 | 45.467 |
| 22200.444 | 44.928 |
| 22300.446 | 44.693 |
| 22400.448 | 44.664 |
| 22500.45 | 44.908 |
| 22600.452 | 44.733 |
| 22700.454 | 44.444 |
| 22800.456 | 44.256 |
| 22900.458 | 44.271 |
| 23000.46 | 44.867 |
| 23100.462 | 43.414 |
| 23200.464 | 43.404 |
| 23300.466 | 43.816 |
| 23400.468 | 44.317 |
| 23500.47 | 44.926 |
| 23600.472 | 45.33 |
| 23700.474 | 45.23 |
| 23800.476 | 45.32 |
| 23900.478 | 45.809 |
| 24000.48 | 46.092 |
| 24100.482 | 45.92 |
| 24200.484 | 45.593 |
| 24300.486 | 45.935 |
| 24400.488 | 45.265 |
| 24500.49 | 45.182 |
| 24600.492 | 45.051 |
| 24700.494 | 45.28 |
| 24800.496 | 45.004 |
| 24900.498 | 44.898 |
| 25000.5 | 44.779 |
| 25100.502 | 44.387 |
| 25200.504 | 44.408 |
| 25300.506 | 44.636 |
| 25400.508 | 44.566 |
| 25500.51 | 44.267 |
| 25600.512 | 44.171 |
| 25700.514 | 44.22 |
| 25800.516 | 44.096 |
| 25900.518 | 43.425 |
| 26000.52 | 43.098 |
| 26100.522 | 42.992 |
| 26200.524 | 42.916 |
| 26300.526 | 41.076 |
| 26400.528 | 42.901 |
| 26500.53 | 40.947 |
| 26600.532 | 40.833 |
| 26700.534 | 40.698 |
| 26800.536 | 40.79 |
| 26900.538 | 41.033 |
| 27000.54 | 41.111 |
| 27100.542 | 41.392 |
| 27200.544 | 41.446 |
| 27300.546 | 41.212 |
| 27400.548 | 41.238 |
| 27500.55 | 40.955 |
| 27600.552 | 41.229 |
| 27700.554 | 41.291 |
| 27800.556 | 41.048 |
| 27900.558 | 41.623 |
| 28000.56 | 41.756 |
| 28100.562 | 41.147 |
| 28200.564 | 41.266 |
| 28300.566 | 40.676 |
| 28400.568 | 41.004 |
| 28500.57 | 41.037 |
| 28600.572 | 41.469 |
| 28700.574 | 41.469 |
| 28800.576 | 41.33 |
| 28900.578 | 40.745 |
| 29000.58 | 40.762 |
| 29100.582 | 40.861 |
| 29200.584 | 40.92 |
| 29300.586 | 40.519 |
| 29400.588 | 40.65 |
| 29500.59 | 40.657 |
| 29600.592 | 39.777 |
| 29700.594 | 40.169 |
| 29800.596 | 40.232 |
| 29900.598 | 40.368 |
| 30000.6 | 40.472 |
| 30100.602 | 40.54 |
| 30200.604 | 40.228 |
| 30300.606 | 40.374 |
| 30400.608 | 40.533 |
| 30500.61 | 40.416 |
| 30600.612 | 40.244 |
| 30700.614 | 40.329 |
| 30800.616 | 40.55 |
| 30900.618 | 40.281 |
| 31000.62 | 40.805 |
| 31100.622 | 39.937 |
| 31200.624 | 40.372 |
| 31300.626 | 40.648 |
| 31400.628 | 40.857 |
| 31500.63 | 40.184 |
| 31600.632 | 40.153 |
| 31700.634 | 40.091 |
| 31800.636 | 40.15 |
| 31900.638 | 40.229 |
| 32000.64 | 40.793 |
| 32100.642 | 40.246 |
| 32200.644 | 40.094 |
| 32300.646 | 40.273 |
| 32400.648 | 39.995 |
| 32500.65 | 39.976 |
| 32600.652 | 39.739 |
| 32700.654 | 39.228 |
| 32800.656 | 40.259 |
| 32900.658 | 39.718 |
| 33000.66 | 42.14 |
| 33100.662 | 42.325 |
| 33200.664 | 39.706 |
| 33300.666 | 40.018 |
| 33400.668 | 40.343 |
| 33500.67 | 39.952 |
| 33600.672 | 39.831 |
| 33700.674 | 40.162 |
| 33800.676 | 39.878 |
| 33900.678 | 40.07 |
| 34000.68 | 39.811 |
| 34100.682 | 40.02 |
| 34200.684 | 40.158 |
| 34300.686 | 40.32 |
| 34400.688 | 40.31 |
| 34500.69 | 40.507 |
| 34600.692 | 40.075 |
| 34700.694 | 40.36 |
| 34800.696 | 40.362 |
| 34900.698 | 40.264 |
| 35000.7 | 40.204 |
| 35100.702 | 40.282 |
| 35200.704 | 40.663 |
| 35300.706 | 40.363 |
| 35400.708 | 40.291 |
| 35500.71 | 40.024 |
| 35600.712 | 40.199 |
| 35700.714 | 39.974 |
| 35800.716 | 39.812 |
| 35900.718 | 39.72 |
| 36000.72 | 39.91 |
| 36100.722 | 40.175 |
| 36200.724 | 40.191 |
| 36300.726 | 40.754 |
| 36400.728 | 40.741 |
| 36500.73 | 40.966 |
| 36600.732 | 40.668 |
| 36700.734 | 39.966 |
| 36800.736 | 39.756 |
| 36900.738 | 39.736 |
| 37000.74 | 39.773 |
| 37100.742 | 39.507 |
| 37200.744 | 39.487 |
| 37300.746 | 39.88 |
| 37400.748 | 39.977 |
| 37500.75 | 39.757 |
| 37600.752 | 39.207 |
| 37700.754 | 39.542 |
| 37800.756 | 39.813 |
| 37900.758 | 39.708 |
| 38000.76 | 39.822 |
| 38100.762 | 38.932 |
| 38200.764 | 38.173 |
| 38300.766 | 38.489 |
| 38400.768 | 38.648 |
| 38500.77 | 38.894 |
| 38600.772 | 39.128 |
| 38700.774 | 39.348 |
| 38800.776 | 38.697 |
| 38900.778 | 38.779 |
| 39000.78 | 38.511 |
| 39100.782 | 38.714 |
| 39200.784 | 38.492 |
| 39300.786 | 38.787 |
| 39400.788 | 38.674 |
| 39500.79 | 39.372 |
| 39600.792 | 39.394 |
| 39700.794 | 39.425 |
| 39800.796 | 39.662 |
| 39900.798 | 39.665 |
| 40000.8 | 39.397 |
| 40100.802 | 39.597 |
| 40200.804 | 39.737 |
| 40300.806 | 39.888 |
| 40400.808 | 39.865 |
| 40500.81 | 39.651 |
| 40600.812 | 39.619 |
| 40700.814 | 39.717 |
| 40800.816 | 39.584 |
| 40900.818 | 39.881 |
| 41000.82 | 39.826 |
| 41100.822 | 39.748 |
| 41200.824 | 39.507 |
| 41300.826 | 39.748 |
| 41400.828 | 39.956 |
| 41500.83 | 39.845 |
| 41600.832 | 39.424 |
| 41700.834 | 39.386 |
| 41800.836 | 39.186 |
| 41900.838 | 39.283 |
| 42000.84 | 39.732 |
| 42100.842 | 39.646 |
| 42200.844 | 39.644 |
| 42300.846 | 39.652 |
| 42400.848 | 39.976 |
| 42500.85 | 39.937 |
| 42600.852 | 39.239 |
| 42700.854 | 39.574 |
| 42800.856 | 39.471 |
| 42900.858 | 39.658 |
| 43000.86 | 39.772 |
| 43100.862 | 40.127 |
| 43200.864 | 39.69 |
| 43300.866 | 40.084 |
| 43400.868 | 39.646 |
| 43500.87 | 40.088 |
| 43600.872 | 40.119 |
| 43700.874 | 40.439 |
| 43800.876 | 40.047 |
| 43900.878 | 40.167 |
| 44000.88 | 40.472 |
| 44100.882 | 40.814 |
| 44200.884 | 40.696 |
| 44300.886 | 40.805 |
| 44400.888 | 41.021 |
| 44500.89 | 40.653 |
| 44600.892 | 40.761 |
| 44700.894 | 41.361 |
| 44800.896 | 40.832 |
| 44900.898 | 40.97 |
| 45000.9 | 40.666 |
| 45100.902 | 40.143 |
| 45200.904 | 40.106 |
| 45300.906 | 40.192 |
| 45400.908 | 40.171 |
| 45500.91 | 40.266 |
| 45600.912 | 40.516 |
| 45700.914 | 40.618 |
| 45800.916 | 40.608 |
| 45900.918 | 40.269 |
| 46000.92 | 40.181 |
| 46100.922 | 40.583 |
| 46200.924 | 41.039 |
| 46300.926 | 40.631 |
| 46400.928 | 40.637 |
| 46500.93 | 40.286 |
| 46600.932 | 40.361 |
| 46700.934 | 40.454 |
| 46800.936 | 40.719 |
| 46900.938 | 40.656 |
| 47000.94 | 40.928 |
| 47100.942 | 40.644 |
| 47200.944 | 40.512 |
| 47300.946 | 40.65 |
| 47400.948 | 40.837 |
| 47500.95 | 41.058 |
| 47600.952 | 41.271 |
| 47700.954 | 41.341 |
| 47800.956 | 41.256 |
| 47900.958 | 41.62 |
| 48000.96 | 41.355 |
| 48100.962 | 40.924 |
| 48200.964 | 41.281 |
| 48300.966 | 41.45 |
| 48400.968 | 41.458 |
| 48500.97 | 41.413 |
| 48600.972 | 41.313 |
| 48700.974 | 40.846 |
| 48800.976 | 40.171 |
| 48900.978 | 40.065 |
| 49000.98 | 40.405 |
| 49100.982 | 40.014 |
| 49200.984 | 39.955 |
| 49300.986 | 40.012 |
| 49400.988 | 39.954 |
| 49500.99 | 39.98 |
| 49600.992 | 40.123 |
| 49700.994 | 40.218 |
| 49800.996 | 40.158 |
| 49900.998 | 39.881 |
| 50001 | 39.89 |
| 50101.002 | 40.077 |
| 50201.004 | 40.551 |
| 50301.006 | 40.119 |
| 50401.008 | 40.278 |
| 50501.01 | 40.388 |
| 50601.012 | 40.506 |
| 50701.014 | 40.333 |
| 50801.016 | 40.535 |
| 50901.018 | 40.889 |
| 51001.02 | 40.8 |
| 51101.022 | 41.1 |
| 51201.024 | 41.48 |
| 51301.026 | 41.19 |
| 51401.028 | 41.323 |
| 51501.03 | 41.298 |
| 51601.032 | 41.203 |
| 51701.034 | 41.307 |
| 51801.036 | 41.134 |
| 51901.038 | 41.043 |
| 52001.04 | 40.981 |
| 52101.042 | 40.936 |
| 52201.044 | 40.926 |
| 52301.046 | 40.887 |
| 52401.048 | 40.76 |
| 52501.05 | 40.81 |
| 52601.052 | 41.132 |
| 52701.054 | 40.669 |
| 52801.056 | 40.926 |
| 52901.058 | 40.782 |
| 53001.06 | 40.656 |
| 53101.062 | 40.616 |
| 53201.064 | 41.079 |
| 53301.066 | 40.707 |
| 53401.068 | 40.616 |
| 53501.07 | 40.576 |
| 53601.072 | 40.453 |
| 53701.074 | 40.675 |
| 53801.076 | 40.685 |
| 53901.078 | 40.91 |
| 54001.08 | 40.849 |
| 54101.082 | 40.415 |
| 54201.084 | 40.225 |
| 54301.086 | 40.143 |
| 54401.088 | 39.9 |
| 54501.09 | 39.613 |
| 54601.092 | 39.699 |
| 54701.094 | 39.657 |
| 54801.096 | 39.963 |
| 54901.098 | 39.815 |
| 55001.1 | 39.406 |
| 55101.102 | 39.671 |
| 55201.104 | 39.673 |
| 55301.106 | 39.762 |
| 55401.108 | 39.871 |
| 55501.11 | 39.573 |
| 55601.112 | 39.675 |
| 55701.114 | 39.805 |
| 55801.116 | 39.996 |
| 55901.118 | 40.372 |
| 56001.12 | 39.968 |
| 56101.122 | 40.201 |
| 56201.124 | 40.434 |
| 56301.126 | 40.326 |
| 56401.128 | 39.913 |
| 56501.13 | 40.007 |
| 56601.132 | 39.052 |
| 56701.134 | 39.605 |
| 56801.136 | 39.641 |
| 56901.138 | 39.719 |
| 57001.14 | 39.511 |
| 57101.142 | 39.471 |
| 57201.144 | 39.525 |
| 57301.146 | 39.47 |
| 57401.148 | 39.138 |
| 57501.15 | 39.624 |
| 57601.152 | 39.081 |
| 57701.154 | 39.098 |
| 57801.156 | 39.07 |
| 57901.158 | 38.745 |
| 58001.16 | 38.846 |
| 58101.162 | 38.653 |
| 58201.164 | 38.823 |
| 58301.166 | 38.858 |
| 58401.168 | 42.895 |
| 58501.17 | 39.016 |
| 58601.172 | 39.258 |
| 58701.174 | 43.157 |
| 58801.176 | 42.841 |
| 58901.178 | 43.081 |
| 59001.18 | 38.905 |
| 59101.182 | 38.793 |
| 59201.184 | 38.774 |
| 59301.186 | 43.685 |
| 59401.188 | 43.151 |
| 59501.19 | 43.162 |
| 59601.192 | 42.152 |
| 59701.194 | 41.242 |
| 59801.196 | 40.9 |
| 59901.198 | 40.659 |
| 60001.2 | 39.747 |
| 60101.202 | 39.475 |
| 60201.204 | 39.627 |
| 60301.206 | 39.541 |
| 60401.208 | 39.659 |
| 60501.21 | 39.416 |
| 60601.212 | 39.485 |
| 60701.214 | 39.55 |
| 60801.216 | 39.502 |
| 60901.218 | 39.017 |
| 61001.22 | 39.384 |
| 61101.222 | 39.194 |
| 61201.224 | 39.345 |
| 61301.226 | 38.97 |
| 61401.228 | 38.905 |
| 61501.23 | 38.449 |
| 61601.232 | 38.551 |
| 61701.234 | 38.806 |
| 61801.236 | 38.692 |
| 61901.238 | 38.626 |
| 62001.24 | 38.557 |
| 62101.242 | 38.355 |
| 62201.244 | 38.185 |
| 62301.246 | 38.271 |
| 62401.248 | 38.587 |
| 62501.25 | 39.222 |
| 62601.252 | 39.544 |
| 62701.254 | 39.872 |
| 62801.256 | 40.146 |
| 62901.258 | 40.285 |
| 63001.26 | 40.37 |
| 63101.262 | 40.539 |
| 63201.264 | 41.01 |
| 63301.266 | 40.878 |
| 63401.268 | 41.266 |
| 63501.27 | 41.369 |
| 63601.272 | 41.365 |
| 63701.274 | 41.527 |
| 63801.276 | 41.754 |
| 63901.278 | 41.352 |
| 64001.28 | 41.435 |
| 64101.282 | 41.579 |
| 64201.284 | 41.571 |
| 64301.286 | 41.234 |
| 64401.288 | 41.344 |
| 64501.29 | 41.046 |
| 64601.292 | 40.482 |
| 64701.294 | 40.199 |
| 64801.296 | 40.071 |
| 64901.298 | 40.093 |
| 65001.3 | 40.199 |
| 65101.302 | 40.036 |
| 65201.304 | 40.203 |
| 65301.306 | 39.926 |
| 65401.308 | 39.731 |
| 65501.31 | 39.361 |
| 65601.312 | 38.923 |
| 65701.314 | 39.297 |
| 65801.316 | 39.146 |
| 65901.318 | 39.405 |
| 66001.32 | 39.056 |
| 66101.322 | 39.276 |
| 66201.324 | 39.173 |
| 66301.326 | 39.004 |
| 66401.328 | 39.232 |
| 66501.33 | 39.287 |
| 66601.332 | 39.367 |
| 66701.334 | 39.53 |
| 66801.336 | 39.308 |
| 66901.338 | 39.677 |
| 67001.34 | 39.306 |
| 67101.342 | 39.339 |
| 67201.344 | 39.103 |
| 67301.346 | 39.573 |
| 67401.348 | 39.113 |
| 67501.35 | 38.818 |
| 67601.352 | 38.679 |
| 67701.354 | 38.75 |
| 67801.356 | 38.272 |
| 67901.358 | 38.403 |
| 68001.36 | 38.007 |
| 68101.362 | 38.132 |
| 68201.364 | 37.842 |
| 68301.366 | 37.936 |
| 68401.368 | 38.195 |
| 68501.37 | 38.26 |
| 68601.372 | 38.116 |
| 68701.374 | 38.008 |
| 68801.376 | 38.205 |
| 68901.378 | 38.508 |
| 69001.38 | 38.574 |
| 69101.382 | 38.38 |
| 69201.384 | 38.054 |
| 69301.386 | 38.172 |
| 69401.388 | 38.366 |
| 69501.39 | 38.784 |
| 69601.392 | 39.024 |
| 69701.394 | 39.396 |
| 69801.396 | 39.833 |
| 69901.398 | 39.482 |
| 70001.4 | 39.668 |
| 70101.402 | 39.235 |
| 70201.404 | 39.726 |
| 70301.406 | 39.928 |
| 70401.408 | 40.385 |
| 70501.41 | 40.578 |
| 70601.412 | 40.619 |
| 70701.414 | 40.535 |
| 70801.416 | 40.831 |
| 70901.418 | 41.134 |
| 71001.42 | 40.56 |
| 71101.422 | 40.846 |
| 71201.424 | 41.267 |
| 71301.426 | 41.598 |
| 71401.428 | 41.864 |
| 71501.43 | 42.132 |
| 71601.432 | 42.197 |
| 71701.434 | 41.902 |
| 71801.436 | 41.753 |
| 71901.438 | 41.879 |
| 72001.44 | 41.828 |
| 72101.442 | 41.647 |
| 72201.444 | 41.268 |
| 72301.446 | 40.612 |
| 72401.448 | 40.095 |
| 72501.45 | 39.541 |
| 72601.452 | 39.816 |
| 72701.454 | 39.09 |
| 72801.456 | 39.206 |
| 72901.458 | 38.66 |
| 73001.46 | 38.528 |
| 73101.462 | 38.389 |
| 73201.464 | 38.795 |
| 73301.466 | 38.347 |
| 73401.468 | 38.328 |
| 73501.47 | 38.342 |
| 73601.472 | 38.642 |
| 73701.474 | 37.954 |
| 73801.476 | 38.274 |
| 73901.478 | 38.772 |
| 74001.48 | 38.958 |
| 74101.482 | 39.485 |
| 74201.484 | 39.202 |
| 74301.486 | 39.141 |
| 74401.488 | 39.084 |
| 74501.49 | 39.778 |
| 74601.492 | 39.995 |
| 74701.494 | 38.198 |
| 74801.496 | 38.486 |
| 74901.498 | 40.035 |
| 75001.5 | 41.044 |
| 75101.502 | 40.813 |
| 75201.504 | 41.634 |
| 75301.506 | 41.274 |
| 75401.508 | 41.742 |
| 75501.51 | 42.117 |
| 75601.512 | 42.207 |
| 75701.514 | 42.341 |
| 75801.516 | 42.226 |
| 75901.518 | 42.223 |
| 76001.52 | 42.951 |
| 76101.522 | 42.548 |
| 76201.524 | 41.778 |
| 76301.526 | 41.457 |
| 76401.528 | 41.041 |
| 76501.53 | 41.273 |
| 76601.532 | 40.9 |
| 76701.534 | 40.431 |
| 76801.536 | 40.235 |
| 76901.538 | 39.876 |
| 77001.54 | 39.563 |
| 77101.542 | 39.254 |
| 77201.544 | 38.871 |
| 77301.546 | 38.496 |
| 77401.548 | 38.241 |
| 77501.55 | 38.081 |
| 77601.552 | 37.944 |
| 77701.554 | 37.966 |
| 77801.556 | 37.819 |
| 77901.558 | 38.014 |
| 78001.56 | 38.486 |
| 78101.562 | 38.248 |
| 78201.564 | 38.485 |
| 78301.566 | 38.224 |
| 78401.568 | 38.722 |
| 78501.57 | 38.914 |
| 78601.572 | 39.058 |
| 78701.574 | 39.537 |
| 78801.576 | 38.685 |
| 78901.578 | 39.132 |
| 79001.58 | 38.663 |
| 79101.582 | 39.362 |
| 79201.584 | 39.799 |
| 79301.586 | 38.553 |
| 79401.588 | 38.399 |
| 79501.59 | 38.103 |
| 79601.592 | 38.279 |
| 79701.594 | 41.342 |
| 79801.596 | 38.025 |
| 79901.598 | 38.225 |
| 80001.6 | 37.981 |
| 80101.602 | 38.021 |
| 80201.604 | 37.851 |
| 80301.606 | 38.214 |
| 80401.608 | 37.884 |
| 80501.61 | 43.379 |
| 80601.612 | 43.335 |
| 80701.614 | 43.364 |
| 80801.616 | 43.299 |
| 80901.618 | 38.784 |
| 81001.62 | 38.832 |
| 81101.622 | 38.922 |
| 81201.624 | 38.696 |
| 81301.626 | 41.934 |
| 81401.628 | 38.653 |
| 81501.63 | 39.004 |
| 81601.632 | 39.118 |
| 81701.634 | 39.03 |
| 81801.636 | 39.164 |
| 81901.638 | 39.226 |
| 82001.64 | 39.414 |
| 82101.642 | 39.405 |
| 82201.644 | 39.116 |
| 82301.646 | 39.198 |
| 82401.648 | 39.152 |
| 82501.65 | 38.69 |
| 82601.652 | 38.304 |
| 82701.654 | 38.843 |
| 82801.656 | 38.52 |
| 82901.658 | 39.033 |
| 83001.66 | 38.801 |
| 83101.662 | 39.076 |
| 83201.664 | 39.058 |
| 83301.666 | 38.999 |
| 83401.668 | 39.379 |
| 83501.67 | 39.028 |
| 83601.672 | 39.184 |
| 83701.674 | 38.771 |
| 83801.676 | 38.887 |
| 83901.678 | 38.193 |
| 84001.68 | 38.602 |
| 84101.682 | 38.829 |
| 84201.684 | 38.609 |
| 84301.686 | 38.669 |
| 84401.688 | 38.561 |
| 84501.69 | 38.341 |
| 84601.692 | 38.28 |
| 84701.694 | 38.244 |
| 84801.696 | 38.581 |
| 84901.698 | 38.702 |
| 85001.7 | 38.619 |
| 85101.702 | 38.866 |
| 85201.704 | 38.301 |
| 85301.706 | 38.288 |
| 85401.708 | 38.406 |
| 85501.71 | 38.349 |
| 85601.712 | 38.738 |
| 85701.714 | 38.788 |
| 85801.716 | 39.026 |
| 85901.718 | 38.857 |
| 86001.72 | 38.976 |
| 86101.722 | 39.148 |
| 86201.724 | 39.432 |
| 86301.726 | 39.489 |
| 86401.728 | 39.196 |
| 86501.73 | 38.936 |
| 86601.732 | 39.204 |
| 86701.734 | 39.113 |
| 86801.736 | 38.943 |
| 86901.738 | 38.464 |
| 87001.74 | 38.513 |
| 87101.742 | 39.019 |
| 87201.744 | 39.272 |
| 87301.746 | 39.13 |
| 87401.748 | 39.255 |
| 87501.75 | 39.146 |
| 87601.752 | 39.446 |
| 87701.754 | 39.139 |
| 87801.756 | 39.146 |
| 87901.758 | 39.168 |
| 88001.76 | 38.861 |
| 88101.762 | 38.634 |
| 88201.764 | 38.61 |
| 88301.766 | 38.357 |
| 88401.768 | 39.154 |
| 88501.77 | 39.123 |
| 88601.772 | 39.132 |
| 88701.774 | 39.11 |
| 88801.776 | 38.701 |
| 88901.778 | 38.304 |
| 89001.78 | 38.434 |
| 89101.782 | 38.476 |
| 89201.784 | 39.252 |
| 89301.786 | 38.981 |
| 89401.788 | 39.018 |
| 89501.79 | 39.247 |
| 89601.792 | 39.495 |
| 89701.794 | 39.437 |
| 89801.796 | 39.477 |
| 89901.798 | 39.691 |
| 90001.8 | 39.314 |
| 90101.802 | 39.054 |
| 90201.804 | 39.21 |
| 90301.806 | 39.262 |
| 90401.808 | 39.157 |
| 90501.81 | 39.348 |
| 90601.812 | 39.953 |
| 90701.814 | 39.578 |
| 90801.816 | 39.264 |
| 90901.818 | 39.001 |
| 91001.82 | 38.926 |
| 91101.822 | 39.113 |
| 91201.824 | 38.768 |
| 91301.826 | 39.032 |
| 91401.828 | 39.065 |
| 91501.83 | 39.315 |
| 91601.832 | 39.475 |
| 91701.834 | 39.685 |
| 91801.836 | 39.58 |
| 91901.838 | 39.163 |
| 92001.84 | 38.785 |
| 92101.842 | 39.6 |
| 92201.844 | 39.417 |
| 92301.846 | 39.164 |
| 92401.848 | 38.797 |
| 92501.85 | 38.837 |
| 92601.852 | 39.385 |
| 92701.854 | 39.507 |
| 92801.856 | 39.271 |
| 92901.858 | 39.291 |
| 93001.86 | 39.132 |
| 93101.862 | 39.927 |
| 93201.864 | 40.081 |
| 93301.866 | 39.472 |
| 93401.868 | 39.807 |
| 93501.87 | 39.43 |
| 93601.872 | 39.955 |
| 93701.874 | 40.057 |
| 93801.876 | 39.974 |
| 93901.878 | 39.656 |
| 94001.88 | 39.367 |
| 94101.882 | 39.341 |
| 94201.884 | 39.213 |
| 94301.886 | 39.532 |
| 94401.888 | 39.228 |
| 94501.89 | 39.385 |
| 94601.892 | 38.985 |
| 94701.894 | 38.795 |
| 94801.896 | 38.749 |
| 94901.898 | 38.762 |
| 95001.9 | 38.591 |
| 95101.902 | 38.224 |
| 95201.904 | 38.202 |
| 95301.906 | 38.533 |
| 95401.908 | 38.749 |
| 95501.91 | 38.563 |
| 95601.912 | 38.422 |
| 95701.914 | 38.558 |
| 95801.916 | 38.551 |
| 95901.918 | 38.795 |
| 96001.92 | 38.433 |
| 96101.922 | 38.325 |
| 96201.924 | 38.854 |
| 96301.926 | 38.701 |
| 96401.928 | 38.5 |
| 96501.93 | 39.127 |
| 96601.932 | 39.042 |
| 96701.934 | 38.697 |
| 96801.936 | 38.515 |
| 96901.938 | 38.667 |
| 97001.94 | 38.77 |
| 97101.942 | 38.54 |
| 97201.944 | 39.119 |
| 97301.946 | 38.857 |
| 97401.948 | 39.204 |
| 97501.95 | 38.9 |
| 97601.952 | 38.699 |
| 97701.954 | 39.33 |
| 97801.956 | 38.753 |
| 97901.958 | 38.979 |
| 98001.96 | 38.85 |
| 98101.962 | 39.291 |
| 98201.964 | 38.913 |
| 98301.966 | 39.272 |
| 98401.968 | 39.658 |
| 98501.97 | 39.335 |
| 98601.972 | 39.172 |
| 98701.974 | 39.032 |
| 98801.976 | 38.869 |
| 98901.978 | 38.415 |
| 99001.98 | 38.991 |
| 99101.982 | 38.56 |
| 99201.984 | 38.546 |
| 99301.986 | 38.709 |
| 99401.988 | 38.8 |
| 99501.99 | 38.613 |
| 99601.992 | 38.476 |
| 99701.994 | 38.65 |
| 99801.996 | 38.704 |
| 99901.998 | 39.567 |
| 100000.008 | 38.982 |
